# Improving mobility and participation of older people with vertigo, dizziness and balance disorders in primary care using a care pathway: feasibility study and process evaluation

**DOI:** 10.1186/s12875-021-01410-2

**Published:** 2021-04-02

**Authors:** Eva Seckler, Verena Regauer, Melanie Krüger, Anna Gabriel, Joachim Hermsdörfer, Carolin Niemietz, Petra Bauer, Martin Müller

**Affiliations:** 1 Centre for Research, Development and Technology Transfer, Rosenheim Technical University of Applied Sciences, Hochschulstraße 1, 83024 Rosenheim, Germany; 2 Institute for Medical Information Processing, Biometry and Epidemiology, Ludwig-Maximilians-Universität München, Marchioninistraße 17, 81377 Munich, Germany; 3 Institute of Sports Science, Leibniz University Hannover, Am Moritzwinkel 6, 30167 Hannover, Germany; 4 Department of Sport and Health Sciences, Technical University of Munich, Georg-Brauchle-Ring 60/62, 80992 Munich, Germany; 5 Faculty for Applied Health and Social Sciences and Centre for Research, Development and Technology Transfer, Rosenheim Technical University of Applied Sciences, Hochschulstraße 1, 83024 Rosenheim, Germany

**Keywords:** Critical pathways, Primary health care, General practitioners, Aged, Vertigo, Dizziness, Physical therapy modalities, Implementation science, Feasibility studies

## Abstract

**Background:**

Community-dwelling older people are frequently affected by vertigo, dizziness and balance disorders (VDB). We previously developed a care pathway (CPW) to improve their mobility and participation by offering standardized approaches for general practitioners (GPs) and physical therapists (PTs). We aimed to assess the feasibility of the intervention, its implementation strategy and the study procedures in preparation for the subsequent main trial.

**Methods:**

This 12-week prospective cohort feasibility study was accompanied by a process evaluation designed according to the *UK Medical Research Council’s Guidance for developing and evaluating complex interventions*. Patients with VDB (≥65 years), GPs and PTs in primary care were included. The intervention consisted of a diagnostic screening checklist for GPs and a guide for PTs. The implementation strategy included specific educational trainings and a telephone helpline. Data for mixed-method process evaluation were collected via standardized questionnaires, field notes and qualitative interviews. Quantitative data were analysed using descriptive statistics, qualitative data using content analysis.

**Results:**

A total of five GP practices (seven single GPs), 10 PT practices and 22 patients were included in the study. The recruitment of GPs and patients was challenging (response rates: GP practices: 28%, PT practices: 39%). Ninety-one percent of the patients and all health professionals completed the study. The health professionals responded well to the educational trainings; the utilization of the telephone helpline was low (one call each from GPs and PTs). Familiarisation with the routine of application of the intervention and positive attitudes were emphasized as facilitators of the implementation of the intervention, whereas a lack of time was mentioned as a barrier. Despite difficulties in the GPs’ adherence to the intervention protocol, the GPs, PTs and patients saw benefit in the intervention. The patients’ treatment adherence to physical therapy was good. There were minor issues in data collection, but no unintended consequences.

**Conclusion:**

Although the process evaluation provided good support for the feasibility of study procedures, the intervention and its implementation strategy, we identified a need for improvement in recruitment of participants, the GP intervention part and the data collection procedures. The findings will inform the main trial to test the interventions effectiveness in a cluster RCT.

**Trial registration:**

Projektdatenbank Versorgungsforschung Deutschland (German registry Health Services Research) VfD_MobilE-PHY_17_003910, date of registration: 30.11.2017; Deutsches Register Klinischer Studien (German Clinical Trials Register) DRKS00022918, date of registration: 03.09.2020 (retrospectively registered).

**Supplementary Information:**

The online version contains supplementary material available at 10.1186/s12875-021-01410-2.

## Background

Vertigo, dizziness and balance disorders (VDB) are frequent complaints of older people [1–4], with a reported prevalence of up to 50% [5–8]. VDB in older persons are a distinct risk factor for falls [2] and even fear of falling may lead to activity restriction and disability [9]. The occurrence of these symptoms is a common reason for consultation in general practice, with a reported consultation prevalence of up to 16% [10]. Due to multifactorial aetiology [8, 11–13], the overutilization of health care in affected patients insufficiently treated in primary care has been shown [14, 15]. Physical therapy is likely to be a valuable component in the management of patients with VDB regarding consequences such as imbalance and falls that result in limited mobility and participation restrictions [16–19]. Despite the sufficient quality of evidence indicating the value of physical therapy for managing VDB, physical therapy seems not to be a standard option in the primary care of patients with chronic VDB in Germany [20].

A care pathway (CPW) is an evidence-based, structured, multi-disciplinary care plan that describes all relevant diagnostic and therapeutic steps in the care of patients with a specific health problem in chronological order; it is used to translate scientific evidence into local practice by considering regional conditions and demands [21, 22]. CPWs might be a promising approach to optimizing the care of older patients with VDB by integrating specific physical therapy interventions and referral guidelines into primary care. We previously developed a multi-disciplinary CPW that aims to improve participation and mobility in older adults with VDB in the primary care setting by offering standardized approaches for general medicine and physical therapy. Since the implementation of complex interventions is a challenging task, the *UK Medical Research Council (MRC) Guidance for the systematic development and evaluation of complex interventions* [23] recommends a feasibility/piloting phase prior to a future definitive trial. Consequently, we aimed to assess our developed intervention in a feasibility study. To understand the process, we conducted a comprehensive process evaluation to investigate its strengths and weaknesses.

Specific objectives were to evaluate:

1. The trial feasibility of the proposed study design (1.1) to explore the recruitment of clusters (general practitioners (GPs)), physical therapists (PTs), and individuals and (1.2) to test the acceptability and eligibility of the outcome measures and data collection procedures;
2. The feasibility, acceptability and usability of the intervention components;
3. The feasibility and acceptability of the implementation strategy by identifying facilitators and barriers in the domains of context and delivery to and response of clusters, PTs, and individuals;
4. The unintended consequences of the processes and outcomes of the intervention and its implementation strategy.

## Methods

### Study design

This prospective cohort feasibility study aimed to simulate the intervention arm of a future cluster RCT (cRCT). It was accompanied by a mixed-method process evaluation to obtain a detailed comprehension of how the intervention works. Since we experienced problems with the recruitment of clusters in the study, we decided to focus on the experimental intervention rather than a control intervention.

Reporting of this study followed the *Consolidated Standards of Reporting Trials* (CONSORT) *statement* extension for pilot and feasibility trials [24] and the *Template for Intervention Description and Replication* (TIDieR) [25].

### Participants and setting

Participants were patients (individuals), GP practices (clusters) and PT practices. We decided not to define a dyad consisting of a GP practice and a PT practice as a cluster, as the patients were free to choose all PTs trained within the study context and therefore did not necessarily opt for the nearest PT practice.

GP practices (clusters) were eligible when the physicians had professional working experience with patients with VDB and statutory health insurance accreditation, which means that a GPs is authorized to treat patients who are compulsorily insured by statutory health insurance, which covers almost 90% of the population. Initially, we considered including only health professionals with at least 3 years of working experience after medical licensure, but due to organizational and availability reasons, we decided not to employ this limitation. GP practices were recruited in the region of southern Bavaria, Germany, and were identified via a database search. The initial invitation to participate was made via telephone call followed by an email and a personal visit for further information.

Eligible patients (individuals) had to be at least 65 years old and had to have consulted with their GP regarding complaints of VDB of any aetiology within the last 3 years. They had to have no legal guardian and appropriate verbal and cognitive command of the German language to give written informed consent, complete the questionnaires and follow verbal and written instructions. Due to the administration of a physical performance test for outcome measurement, the patients also had to be able to walk 10 m (with or without walking aids). Patients were excluded from the study if in-patient hospital treatment was required. After giving informed consent, the recruited GPs were asked to identify eligible patients based on a provided list of inclusion criteria by searching their practice software using *International Statistical Classification of Diseases and Related Health Problems* (ICD) codes or free text searches (see Additional file 1 for manual for the recruitment of patients) and to recruit them by sending informational documents by postal mail. With this recruitment procedure we intended to simulate a baseline assessment before randomization for a planned future cRCT.

Local PT practices were identified based on the GPs’ recommendations and additional geographic screening. PTs were invited to take part in the study via telephone call followed by an email with further information. The same inclusion criteria for GPs applied for PTs.

### The intervention

The intervention is a CPW to improve participation and mobility in older adults with VDB in the primary care setting by offering standardized approaches for general medicine and physical therapy.

#### Development

The development of the CPW and its implementation strategy systematically combined existing evidence from previous research with a co-creation approach considering different perspectives. Health professionals, patients and experts in the field were systematically involved. Further information about the intervention, its development and the modelling process of intervention strategies will be published elsewhere in detail.

#### Content and implementation strategy

The developed multi-disciplinary CPW is a paper-based algorithm providing a structured illustration of all steps of the patient’s path; it consists of two main components:

1. A checklist for diagnostic screening for GPs that describes evidence-based diagnostics, treatment and referral options and specific time lines for follow-ups.
2. An evidence-based guide for clinical reasoning and treatment of VDB for PTs that includes evidence-based patient information (leaflets with home exercises) and informational flyers (on symptom control and frequently asked questions about specific conditions), as a referral to physical therapy is a relevant option for patients with VDB.

The checklist and the guide are not available since they have not yet been evaluated for effectiveness and safety.

**Fig. 1:**
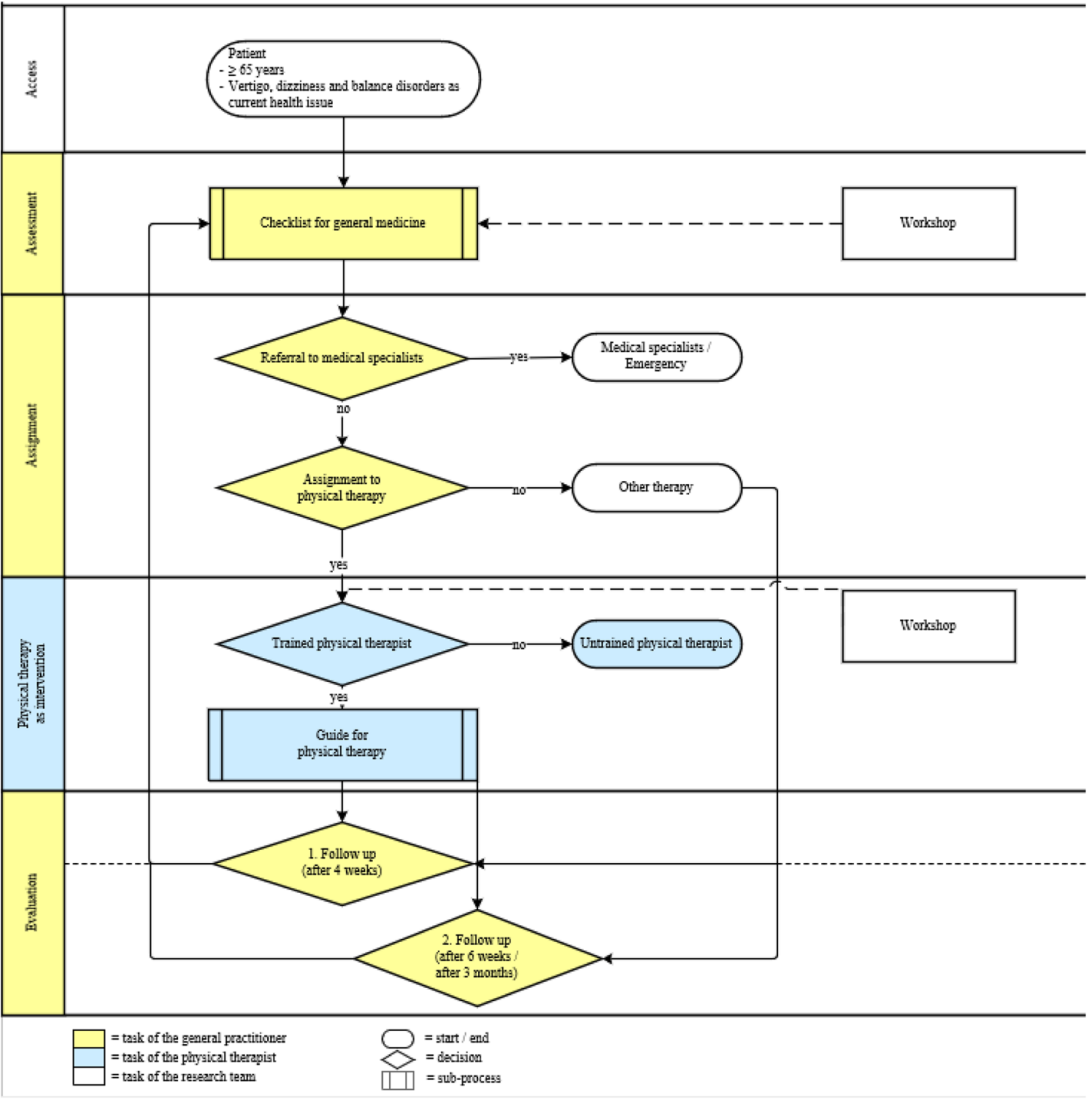
Overview of the patient’s path in the intervention

The relationship between the CPW components is illustrated in Fig. 1.

**Fig. 2:**
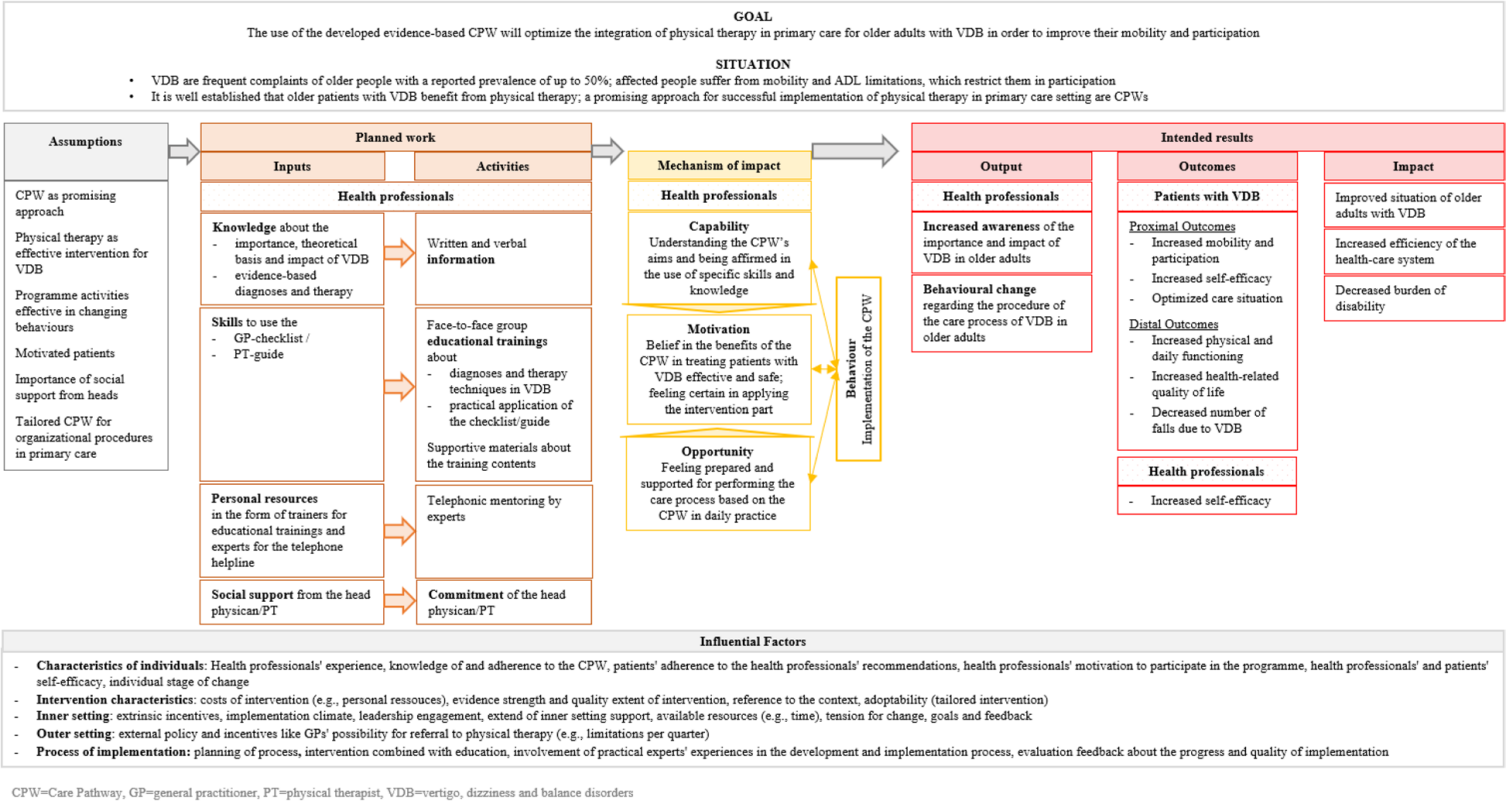
Logic model of the CPW

We developed a logic model (see Fig. 2) describing a mechanism of change using the central model of the *Behaviour Change Wheel* (BCW), the *Capability-Opportunity-Motivation-Behaviour* (COM-B) model [26]. In addition, we considered potential influencing factors classified according to the five main elements of the *Consolidated Framework of Implementation Research* [27].

The key components of the implementation strategy were face-to-face educational group trainings for the GPs (90 min) and for the PTs (one day) containing demonstrations of required skills, do-it-yourself-elements with feedback and instructions for the intended application each part of the corresponding CPW. The participants received additional written information. The training for the GPs was held by a neurologist, and the training for the PTs was held by a specialist PT. Both trainings included a brief information about the study background and logistics provided by the research team. Participation in these training sessions was free of charge and included a qualification certificate. A telephone mentoring helpline for the GPs was provided by an oto-neurologist who was also the co-developer of the checklist and administered the training. A telephone mentoring helpline for the PTs was provided by a member of the research team, who is an experienced PT.

The health professionals obtained a certificate for study participation to display in their practice as well as a payment per treated study patient (GPs: 40€; PTs: 20€).

### Outcomes and data collection procedures

We collected patient data for the primary and secondary outcomes at three measurement points: at baseline (T0), after 6 weeks (T1) and after 12 weeks (T2). The patients could opt to participate in the data collection in their homes or at a study centre visit. Prior to conducting this trial, we pre-tested all documents on two volunteers.

An overview of used outcome assessments and timeline is shown in Table 1.

**Table 1:** Overview of used outcome assessments and timeline

Outcomes	Data collection procedures/assessments		Study period				
			Enrolment	Time of data collection			Close-out
			*Pre T0*	*T0*	*T1*	*T2*	*Post T2*
**Primary outcome**							
- Impact of dizziness on activities of daily living	Dizziness Handicap Inventory (DHI)			X	X	X	
**Secondary outcomes**							
- Static and dynamic balance	Mini-Balance Evaluation Systems Test (miniBEST)			X	X	X	
- Health-related quality of life	EuroQol 5-dimension 5-level (EQ-5D-5L)			X	X	X	
- Daily-life physical activity profile	Actigraphy (StepWatch4, Move4)			X	X	X	
- Types of physical activity in daily life	International Physical Activity Questionnaire (IPAQ)		X	X^a^	X^a^	X^a^	
- Time and types of physical activity; daily time spent moving, sitting, lying; and occurrence of VDB	Physical activity diary			X	X	X	
**Process evaluation**							
- Characteristics of participants	Standardized questionnaire on sociodemographic data		X				
- Structural practice data of GP and PT practices	Standardized questionnaire on structural practice data based on the QCPC		X				
- Trial feasibility - Feasibility of the intervention components - Feasibility of the implementation strategy	Research team	Field notes by the research team		X	X	X	
		Field notes by the study assistant after each measurement appointment		X	X	X	
	GPs	Group interview with GPs			X		
		Individual interview with GPs	X				
		Standardized questionnaire on the recruitment process	X				
		Standardized evaluation forms for the educational trainings	X				
		Field notes on contact with GPs via telephone or email	X	X	X	X	X
		Field notes by GPs^c^		X	X	X	
	PTs	Individual interviews with PTs					X
		Standardized evaluation forms for the educational training	X				
		Field notes by PTs^d^		X	X	X	
		Field notes on contact with PTs via telephone or email	X	X	X	X	X
	Patients	Individual interviews with patients			X		X
		Patients’ cancellation forms	X				
		Standardized evaluation forms after each questionnaire	X	X	X	X	
		Field notes by the patients^b^		X	X	X	
		Field notes on contact with patients via telephone or email	X	X	X	X	X

*GP* general practitioner, *PT* physical therapist, *QCPC* Questionnaire of Chronic Illness Care in Primary Care, *VDB* vertigo, dizziness and balance disorders

^a^ one week after measurement point

^b^ Patients’ field notes in free text option in physical activity diary

^c^ GPs’ field notes in form of a completed checklist including a free text option

^d^ PTs’ field notes in form of a completed guide including a free text option and treatment documentation

#### Primary outcome

The impact of VDB on the Activities of Daily Living, as the primary outcome, was assessed by the *Dizziness Handicap Inventory* (DHI) [28].

#### Secondary outcomes

The secondary outcomes were balance, measured by the *Mini-Balance Evaluation Systems Test* (miniBEST) [29], and health-related quality of life assessed by the *EuroQol 5-dimension 5-level* (EQ-5D-5L) questionnaire [30]. Table 1 displays all secondary outcomes (patient-reported outcomes and performance tests). For the objective assessment of physical activity profiles, the patients were asked to wear two different activity sensors: (1) Move4 (Movisens GmbH, Germany), attached at the thigh with adhesive tape, and (2) StepWatch4 (modus health llc, USA), worn on the ankle with a strap. The patients were asked to wear both sensors simultaneously for five consecutive days within the week following T0, T1 and T2 to collect information about their daily life physical activity. In addition, physical activity was quantitatively assessed by the *International Physical Activity Questionnaire* (IPAQ) [31]. Furthermore, the patients were required to maintain a combined physical activity/dizziness-diary while wearing the sensor.

#### Data collection procedures

At baseline, the patients completed the patient-reported outcome questionnaires together with a study assistant; at follow-up, the patients were asked to complete the questionnaires by themselves, but assistance was provided on request. The completition of the miniBEST performance test and the distribution and attachment of the sensors were done in a personal appointment with the patient and the study assistant. The results of the miniBEST and DHI were shared with the treating PTs to inform further therapy planning.

#### Process evaluation

The process evaluation followed the respective *UK MRC Guidance for process evaluation of complex interventions* covering the domains of implementation, mechanism of impact and context [32] along with the *Framework for design and reporting of process evaluation* by Grant et al. [33]. The process evaluation was structured according to the following domains: recruitment of clusters and individuals, context, delivery to and response of clusters and individuals and unintended consequences. We did not consider effectiveness domain, because we did not aim to estimate any treatment effects. Due to the short duration of the study, we also did not consider the maintenance domain. We additionally observed the performance and feasibility of the outcome measures and data collection procedures.

For data collection, we used continuous field notes; standardized questionnaires for the study participants; semi-structured individual telephone interviews with the GPs, patients and PTs; and a face-to-face group interview with the GPs and checklist developers. The interviews were conducted by members of the research team (ES, VR), and group discussion was moderated by both researchers.

**Fig. 3:**
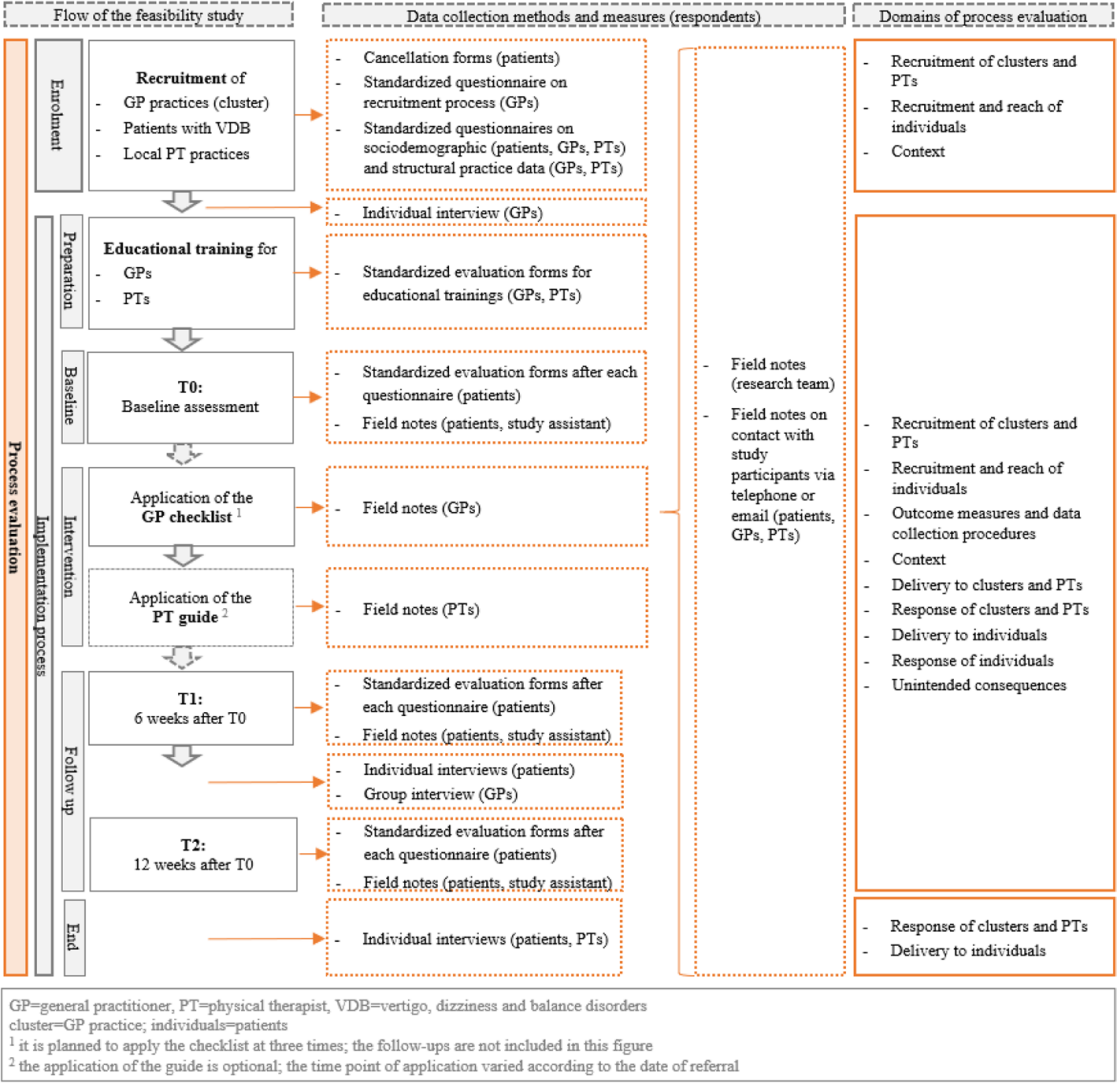
Flow diagram of the process evaluation alongside the feasibility study

For an overview of the procedure of the process evaluation alongside the feasibility study see Fig. 3.

Detailed information about data collection methods in the different domains and time points can be taken from Additional file 2.

#### Trial feasibility

##### Recruitment of clusters and PTs

The recruitment of health professionals was assessed before and during the intervention. Reasons for study participation were documented by personal interviews. The recruitment procedure and retention rate including reasons for early study termination were investigated via continuous field notes. The flow of recruitment and the reach of the intervention were documented using protocols. The participants were asked about their satisfaction with the recruitment via personal interviews. To assess sociodemographic information and structural practice data, we used a questionnaire based on the *Questionnaire of Chronic Illness Care in Primary Care* (QCPC) [34].

##### Recruitment and reach of individuals

The recruitment of individuals and intervention reach among patients were assessed before and during the intervention. To investigate the recruitment procedure, we performed personal or telephone interviews with the patients and GPs, used a standardized questionnaire on the recruitment procedure used by the GPs and analysed field notes. To evaluate the patients’ motivation, we collected information about their reasons for participation in interviews and for their non-participation using a short questionnaire. The flow of recruitment of individuals and intervention reach among patients was documented using recruitment protocols. To evaluate the responses, we asked about the patients’ satisfaction with recruitment in the interviews. Sociodemographic information was collected at baseline via a standardized questionnaire.

The retention rate including reasons for early study termination in all participants was documented.

##### Outcome measures and data collection procedures in the patients

The utilization of the outcome measures and the performance of data collection procedures in the patients were assessed during the intervention. To evaluate delivery, protocol deviations and missing data were documented. The patients’ responses regarding measurement procedures, satisfaction with organizational aspects and effort required for study participation were evaluated by analysing the interviews and the contact and field notes. To assess the feasibility of the questionnaires, we asked patients to complete a supplemental evaluation form about difficulties and time consumption.

##### Outcome measures and data collection procedures in the clusters and PTs

The acceptability and eligibility of the selected outcome measures and data collection procedures were determined during the intervention via field notes, interviews and contact with the health professionals to evaluate their responses regarding the procedures, study logistics, effort and feasibility of study participation in daily practice.

##### Feasibility of the intervention components and implementation strategy

The evaluation of the intervention components and its implementation strategy included the assessment of context; delivery to and response of the clusters, PTs, and individuals; and unintended consequences. The data were collected prior to, during and after the intervention to appraise changes over time.

##### Context

Information about the GP and PT practices was collected by a questionnaire based on the QCPC [34] immediately after study enrolment. Contextual factors in terms of barriers and facilitators in the implementation of the interventions were assessed through a group interview with the GPs, individual interviews with the PTs and patients and the analysis of field notes.

##### Delivery to and response of clusters and PTs

The delivery of the intervention to health professionals was assessed during the intervention via interviews and field notes. The health professionals’ responses about the intervention and its integrability into daily practice, including difficulties in delivery, experiences within the implementation process and adaptations were assessed during and after the intervention. Standardized evaluation forms were used to evaluate educational trainings. Additionally, we analysed the interviews, field notes and contact notes. The support offered by the helplines (e.g., satisfaction and use) was assessed via interviews and the analysis of the contact field notes. The health professionals’ satisfaction with the intervention, their adherence to it and any adjustments they made were evaluated in interviews and via the analysis of notes from contact with the participants and field notes, including the completed checklists/guides. Analysis of field notes was also used to evaluate deviations from the implementation protocol and attendance. Attitude and behaviour changes of the health professionals in daily practice and their experiences during the implementation process were assessed through interviews and field notes.

##### Delivery to and response of individuals

The delivery of the intervention components to the patients was evaluated during and after the intervention through interviews with the patients and health professionals and contact and field notes, including a comparison with the completed checklists/guides. Telephone interviews with the target group were used to assess the patients’ experience of and response to the intervention, including their adherence and behavioural change.

##### Unintended consequences

Unintended consequences of the process and outcomes of the intervention and its implementation strategy were assessed during the intervention through interviews with the participants and field notes by the research team.

### Sample size

A sample size calculation was not performed since we did not aim to estimate any treatment effects. The analysis must therefore be considered exploratory. Based on pragmatic considerations and to obtain sufficient information about the feasibility and acceptance of the intervention and the feasibility of the study procedures, we planned to include five GP practices, each with five to 10 patients, in the study.

### Data analysis

For the analysis of the assessment instruments, standardized questionnaires and some of the documentary data, we entered the data in a secure, web-based software platform designed to support data capture for research studies named Research Electronic Data Capture (REDCap) and used descriptive statistics.

Statistical analysis of the patient data was performed using R statistical software [35]. Since the focus of this study was on feasibility, we did not calculate statistical significance, as is often erroneously done in feasibility studies [36]. The study assistant who assessed and entered the data was not involved in the analysis.

The qualitative interviews were audio-recorded and transcribed verbatim according to the rules proposed by Kuckartz [37] with F4 transcription software, and the field notes were used to provide context in this process. Analysis was conducted by two researchers (ES, VR) independently using MAXQDA software [38] following the process of content analysis according to the concept of qualitative description [39, 40]. If necessary, any disagreements between the coders were discussed with a third researcher (MM). In terms of quality assurance, the group interview participants were offered the opportunity to verify and modify the results. Analysis of the notes from contact with study participants via the telephone helpline, the hotline or email and analysis of parts of the continuous field notes and physical activity diaries were also conducted qualitatively.

Sensor-based activity data were evaluated in a multi-step process. The pre-processing of sensor-based activity data was performed using the software provided by the manufacturers, i.e., SensorManager (Movisens) and StepWatch 4 RE (StepWatch). For both sensors, recorded accelerometer data were aggregated into 1 min-epochs for the whole period of data recording. Based on this approach, for each time epoch, the following parameters were extracted: steps (Movisens and StepWatch) and activity class (sitting/lying, standing, and moving, for Movisens only). All subsequent data processing was performed using Excel (Microsoft Corporation, Redmond, WA, USA). In the first step, for each patient, each measurement point and each parameter, the data were pooled in 24-h periods, i.e., recording days. Based on this approach, the following parameters were calculated for each recording day: difference in the number of recorded steps between the two sensors, i.e., steps_Movisens_ - steps_StepWatch_; the share of each activity class, expressed as the percentage of the recording day; and the mean duration spent consecutively in one activity class, hereafter referred to as the mean bout length. Subsequently, valid recording days were identified by the following factors [41–44]: the patients had to wear the sensor for at least 10 hours and walk at least 200 steps. Only patients with at least four valid recording days were included in further analytical steps. Next, for each patient and each of the above-mentioned parameters, the mean across all valid recording days was calculated. To interpret quantitative differences between activity sensors, the physical activity diaries were used for qualitative assessment of the patients’ physical activity. In addition, the main outcome measure of the IPAQ, i.e., metabolic equivalent task minutes per week (METmin/week), was included in the analysis. Statistical analysis was performed using SPSS Statistics 23 (IBM Corp., Armonk, NY, United States).

## Results

### Trial feasibility

#### Recruitment of clusters

The recruitment of clusters took place between February and April 2019 and was time consuming due to the GPs’ limited availability, and issues in receiving the information via email; the use of fax was found to be more practical. Since most GPs cancelled the initially planned information event for time reasons, we visited each practice to provide further information (mean duration: 22 min). The GPs characterized the information documents as complete and sufficient.

For further information and an overview of barriers and facilitators subdivided in all domains see Additional file 3.

A total of 18 GP practices were approached via telephone calls, and nine GP practices of interest were visited on site; five practices with a total of seven GPs agreed to take part. See Table 2 for further details.

**Table 2:** Characteristics of the health professionals at baseline

	GPs (*n* = 7)	PTs (*n* = 11)
Age, *mean (range)*	54.6 (37.0–66.0)	41.3 (24.0–61.0)
Sex, *n female (%)*	1 (14.3)	9 (81.8)
Years of professional activity, *mean (range)*	21.1 (7.0–35.0)	18.3 (1.0–40.0)

*GP* general practitioner, *PT* physical therapist

**Fig. 4:**
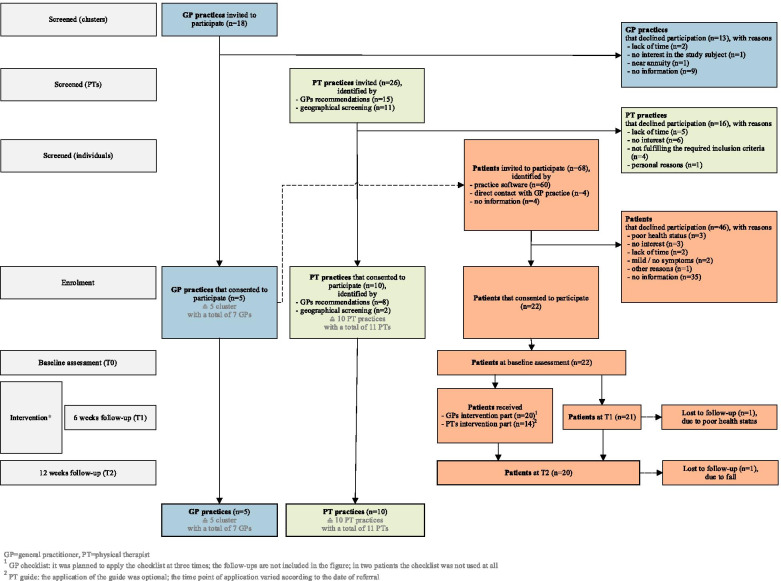
Flow of participants through the feasibility trial

In most cases, reasons for non-participation were not given (for further information see Fig. 4). Reasons for participation mostly included a perception of the topic as interesting and of practical relevance, the desire to improve treatment quality through a structured approach, and the desire for intra-professional exchange and a general interest in research projects.

All clusters completed the study. For flow of participants through this study see Fig. 4.

#### Recruitment of PTs

The telephone requests to PT practices and internal forwarding of information proceeded without issues. The PTs were satisfied with the recruitment approach including the structure, content and the extent of the information material.

The recruitment of PT practices took place between April and May 2019. A total of 10 PT practices out of the 26 approached agreed to participate and completed the study (see Fig. 4). The PTs’ mean age was 41.3 years, and most of them were women (82%) (for further information see Table 2).

Reasons for non-participation were a lack of interest and time (for further information see Fig. 4), whereas reasons for participation were a perception of the topic as interesting and of practical relevance, the chance to improve quality, and an interest in educational trainings and in research projects in general.

For further information and an overview of barriers and facilitators subdivided in all domains see Additional file 3.

#### Recruitment and reach of individuals

Several problems in the implementation of the intended recruitment approach for patients occurred since there was a considerable delay in the GPs’ initiation of recruitment in spite of repeated reminders. It was difficult for the GPs to apply the inclusion criteria and some invited younger patients (*n* = 2) and those with cognitive impairment (*n* = 1). Hence, the initial planned recruitment period was extended by 3 months. It was noted that the timing was unfavourable, e.g., due to holiday season.

Eighty-eight percent of the potential eligible patients were identified via practice software (as planned), and 6% were invited by direct contact in a GP practice.

*“I think that is always much more convincing for the patient than if he somehow gets a letter. [...] That is why it would have been the natural course of action for me to give it [the study information] to him immediately.”* (GP, 45 years)

*“A kind of one-pager I have at my desk […] where I quickly have the essential points ready to tell the patient what to expect. So, in the next step, if he shows interest, I can simply give him the whole thing, because the difficulty then was to change the daily routine and quickly convey the five or six important points of the study to him.”* (GP, 57 years)

An additional person was needed to help with the time-consuming search via practice software. One GP assigned an office assistant to inform the potential participants about the study by telephone before sending the documents.

The patients were satisfied with information document**s** regarding their comprehensibility, content and extent, but problems in readability occurred due to visual impairment. During the group discussion with the GPs, it was suggested that patients should receive an additional sheet summarizing the most important information.

The GPs identified 68 patients (60 via practice software, 4 through direct contact, and 4 missing data) between May and September 2019. A total of 46 declined participation, and only 24% sent back the cancellation form giving reasons such as a poor health status or no interest (for further information see Fig. 4). A total of 22 patients (32%) consented to participate (range: 3–8 per practice), which was below the planned number of 25 to 60 patients. The GPs suggested the reasons for the poor willingness to participate were the high expenditure of time and work overload involved in study participation, concerns about devices and some patients’ acceptance of their VDB symptoms as given and unchangeable .

*“Especially with these patients, who have been complaining about dizziness for a long time, the willingness to take part and to take on […] a longer examination, then also the announcement that someone is coming to them or that they should possibly go to Rosenheim […] [the participation] is suddenly low. I think that if I had said, ‘Look, I have a pill here, take it and then we will see how it gets better’ - then I would have had no problems.”* (GP, 66 years)

The reasons given by the participants for participation were predominantly personal psychological strain due to VDB symptoms, the hope of improving their own situations or those of others, and general interest.

For further information and an overview of barriers and facilitators subdivided in all domains see Additional file 3.

The patients’ mean age was 78.7 years; most of the patients were women (64%), and four had been rated as having a level of care dependency by expert raters of the medical service of the German statutory health insurance system (0 = “minor”, 1 = “considerable”, 2 = “severe”, 3 = “most severe”; level 2: *n* = 3, level 3: *n* = 1). Half of the patients had received help from family members, friends, relatives or neighbours, and one person had received care from a home care nursing service within the last 4 months. For further information of the patient characteristics see Table 3.

**Table 3:** Characteristics of the patients at baseline

Cluster	C01	C02	C03	C04	C05	Total
General practitioners, *n (%)*	1 (14.3)	1 (14.3)	1 (14.3)	1 (14.3)	3 (42.8)	7 (100.0)
Patients, *n (%)*	4 (18.2)	8 (36.4)	4 (18.2)	3 (13.6)	3 (13.6)	22 (100.0)
Age, *mean (range)*	72.5 (65.0–79.0)	81.3 (73.0–88.0)	78.0 (75.0–80.0)	79.0 (77.0–81.0)	81.0 (80.0–83.0)	78.7 (65.0–88.0)
Woman, *n (%)*	3 (75.0)	5 (62.5)	2 (50.0)	1 (33.3)	3 (100.0)	14 (63.6)
Due to the health status, assistance was received within the last 3 months, via, *n (%)*						
Care by a home care nursing service	0 (0)	0 (0)	0 (0)	0 (0)	1 (33.3)	1 (4.5)
Paid domestic help	0 (0)	1 (12.5)	2 (50.0)	0 (0)	1 (33.3)	4 (18.2)
Help from family members, friends, relatives or neighbours	2 (50.0)	4 (50.0)	2 (50.0)	1 (33.3)	2 (66.7)	11 (50.0)
Areas where assistance from other people is usually needed, *n (%)*						
Dressing and undressing	1 (25.0)	2 (25.0)	1 (25.0)	0 (0)	0 (0)	4 (18.2)
Body care	1 (25.0)	1 (12.5)	1 (25.0)	0 (0)	1 (33.3)	4 (18.2)
Get up	1 (25.0)	0 (0)	0 (0)	0 (0)	0 (0)	1 (4.5)
Food and drink	0 (0)	1 (12.5)	2 (50.0)	0 (0)	0 (0)	3 (13.6)
Walking	1 (25.0)	3 (37.5)	1 (25.0)	0 (0)	0 (0)	5 (22.7)
Domestic help	2 (50.0)	4 (50.0)	3 (75.0)	0 (0)	2 (66.7)	11 (50.0)
Shopping	2 (50.0)	5 (62.5)	2 (50.0)	0 (0)	1 (33.3)	10 (45.5)
Takeover of driving services	1 (25.0)	6 (75.0)	3 (75.0)	1 (33.3)	1 (33.3)	12 (54.5)
Drug intake	0 (0)	5 (62.5)	3 (75.0)	0 (0)	2 (66.7)	10 (45.5)
Other	1 (25.0)	1 (12.5)	0 (0)	1 (33.3)	0 (0)	3 (13.6)
Level of care, *n (%)*	1 (25.0)	1 (12.5)	2 (50.0)	0 (0)	0 (0)	4 (18.2)
Level 0	0 (0)	0 (0)	0 (0)	0 (0)	0 (0)	0 (0)
Level 1	0 (0)	0 (0)	0 (0)	0 (0)	0 (0)	0 (0)
Level 2	0 (0)	1 (12.5)	2 (50.0)	0 (0)	0 (0)	3 (13.6)
Level 3	1 (25.0)	0 (0)	0 (0)	0 (0)	0 (0)	1 (4.5)

No missing values

Overall, 20 patients completed the trial. Two patients dropped out, one due a poor health status and one due to dizziness and subsequent hospitalization (see Fig. 4).

#### Outcome measures and data collection procedures

##### Data collection in the patients

The majority of the participants preferred data collection to take place in their homes due to their mobility restrictions and health status, and only three patients opted for assessment in the study centre.

Since most patients estimated the general effort of study participation to be rather low or even non-existent, the duration of the measurement appointments was satisfactory for them.

In some patients (T0: *n* = 8; T1: *n* = 6; T2: *n* = 2) a relative was present during the measurement.

The patients rated the difficulty of the questionnaires as simple (mean: 2.0; coding: 1 = “very simple”, 2 = “simple”, 3 = “difficult”, 4 = “very difficult”, 5=“impossible without aid”), but some patients needed support from relatives or the study assistant. The patients had the most problems with the IPAQ. The number of missing values in evaluation forms (total blank questionnaires: *n* = 9; questionnaires with a single missing item: *n* = 1) limited the interpretability, while the response rates for the DHI and EQ-5D-5L were 100%. For further information about the results of the standardized evaluation forms see Table 4.

**Table 4:** Results of the standardized evaluation forms for the patients’ questionnaires (DHI, EQ-5D-5L, IPAQ)

	T0 post (1 week)	T1 (6 weeks/7 weeks^a^)			T2 (12 weeks/13 weeks^a^)		
	IPAQ (*n* = 20)	DHI (*n* = 21)	EQ-5D-5L (*n* = 21)	IPAQ^a^ (*n* = 15)	DHI (*n* = 20)	EQ-5D-5L (*n* = 20)	IPAQ^a^ (*n* = 18)
Independent completion possible, *n (%)*	9 (45.0)	14 (66.7)	12 (57.1)	8 (53.3)	16 (80.0)	14 (70.0)	7 (38.9)
Dependent completion with, *n (%)*	11 (55.0)	7 (33.3)	9 (42.9)	7 (46.7)	4 (20.0)	6 (30.0)	11 (61.1)
Relative	5 (25.0)	3 (14.3)	4 (19.0)	1 (6.7)	2 (10.0)	3 (15.0)	3 (16.7)
Acquaintance	0 (0)	0 (0)	0 (0)	0 (0)	0 (0)	0 (0)	0 (0)
Study assistant	6 (30.0)	4 (19.0)	5 (23.8)	6 (40.0)	1 (5.0)	3 (15.0)	7 (38.9)
Difficulty of completition^b^, *median (range)*	2.0 (1.0–5.0)	2.0 (1.0–5.0)	2.0 (1.0–5.0)	2.0 (1.0–5.0)	2.0 (1.0–5.0)	2.0 (1.0–5.0)	2.0 (1.0–5.0)
Time (minutes) of completion, *mean (range)*	13.2 (3.0–60.0)	9.0 (3.0–30.0)	8.4 (2.0–20.0)	11.5 (1.0–30.0)	10.1 (3.0–30.0)	8.1 (2.0–22.0)	9.9 (2.0–30.0)

*EQ-5D-5L* EuroQol 5-dimension 5-level, *DHI* Dizziness Handicap Inventory, *IPAQ* International Physical Activity Questionnaire

^a^ IPAQ measurement times: T0 post (1 week), T1 (7 weeks), T2 (13 weeks)

^b^Coding: 1 = “very simple”, 2 = “simple”, 3 = “difficult”, 4 = “very difficult”, 5 = “impossible without aid”

Missing values: IPAQ: total blank questionnaires T0 (*n* = 1), T1 (*n* = 6), T2 (*n* = 2); single missing item T0 (*n* = 1)

Most participants rated the miniBEST as feasible, but some felt insecure depending on their condition on a particular day or any physical handicaps. Barriers to the performance of the miniBEST in the patients’ homes were narrow rooms and potential stumbling blocks, but the study assistants’ basic qualifications as PTs were an advantage in terms of safety.

The results of the DHI, EQ-5D-5L, IPAQ and miniBEST during the study process of intervention implementation are presented in Table 5. Due to the high number of missing values, no detailed analysis of IPAQ is given in Table 5.

**Table 5:** Results for the primary and secondary outcomes during the study

	Pre T0	T0: baseline^a^	T1: 6 weeks^a^	T2: 12 weeks^a^
	(*n* = 22)	(*n* = 22)	(*n* = 21)	(*n* = 20)
DHI, *median, (range)*	–	38.0 (4.0–84.0)	38.0 (12.0–82.0)	39.0 (6.0–80.0)
EQ-5D-5L, *mean (range)*				
Health state index	–	2.0 (1.6–2.5)	2.1 (1.8–2.6)	2.0 (1.5–2.5)
VAS	–	65.9 (30.0–90.0)	67.6 (20.0–90.0)	59.9 (10.0–90.0)
miniBEST, *median (range)*	–	17.5 (7.0–27.0)	20.0 (12.0–25.0)	19.0 (11.0–27.0)
IPAQ, *mean (range)*	3523.6 (66–12,798)	5793.4 (198–17,598)	4495.8 (146–16,160)	1730.8 (198–4377)

*DHI* Dizziness Handicap Inventory; coding: 0 = “no”, 2 = “sometimes”, 4 = “yes”; missing values: T0 (*n* = 1, item = 1), T1 (*n* = 1, item = 5), T2 (*n* = 1, item = 4)

*EQ-5D-5L* EuroQol 5-dimension 5-level; coding health state index (see distinct item descriptions): 1 = “no problem”, 2 = “slight problem”, 3 = “moderate problem”, 4 = “severe problem”, 5 = “extreme problem”; no missing values

*miniBEST* Mini Balance Evaluation Systems Test; coding (see distinct item descriptions): 0 = “not possible”, 1 = “medium”, 2 = “normal”; no missing values

*IPAQ* International Physical Activity Questionnaire; coding: metabolic equivalent task minutes per week (METmin/week), missing values: preT0 (*n* = 1), T0 (*n* = 5), T1 (*n* = 5), T2 (*n* = 8)

*VAS* visual analogue scale

^a^ one week after measurement point (IPAQ)

The rate for the use of both sensors was rather high (T0: 82%, T1: 86%, T2: 80%), and the patients mostly wore the devices without experiencing any restrictions in daily life, indicating good acceptance. While wearing the StepWatch4, the patients reported the device sliding down, itching, skin irritations and mild oedema and skin irritation. The Move4 required less patient compliance, as this sensor did not need to be removed and replaced by the patients (e.g., before and after taking a shower) during the week of data recording, and allowed better data handling and processing. The lower demand of this sensor might have led to a higher number of obtained valid recording days for the Move4 vs. the StepWatch sensor. Qualitative analysis of the physical activity diary entries suggests that the Move4 sensor better represented differences in physical activity levels within the patients. Thus, further outcomes will be reported only for the Move4 sensor. On average, the eight patients with valid data sets across all three time points took 6148 steps per day at T0, 5482 steps per day at T1 and 5306 steps per day at T2. Analysis of the patients’ activity patterns revealed that the patients spent most of their time sedentary, i.e., sitting, lying or standing. This observation held true for the percentage share of sedentarism compared to that of activity, as well as for the bout length of sedentary phases (see Table 6). Importantly, while the total step count was within the range of that reported in other studies [45], the proportion and bout length of sedentary phases were substantially higher than those of healthy persons of the same age [46].

**Table 6:** Activity pattern in percent of time of the day spent in each class and mean bout length

Activity class		T0	T1	T2
Sitting/lying	*Proportion,*	74%	69%	72%
	*mean bout length*	30.1 min	38.2 min	35.8 min
Standing	*Proportion,*	2%	9%	5%
	*mean bout length*	1.4 min	2.9 min	1.3 min
Moving	*Proportion,*	6%	6%	6%
	*mean bout length*	2.0 min	1.8 min	1.6 min

Please note that the remaining percent of the day was classified as non-wear time

The participants evaluated the physical activity diary as understandable but also as time consuming.

“*I have entered this once every hour. I do not do that anymore. If I am completely honest, I calculate that as an average. When I am on the road or out for a walk, I can of course record it exactly. But how much I walk or sit around at home is more or less estimated.”* (Patient, 77 years).

The rate of completition the diary was rather high (T0: 91%, T1: 81%, T2: 90%), and reasons for refusal were overload or an inability to complete it without assistance, e.g., due to visual impairment or writing problems. Despite the different levels of accuracy of the described activities, the diary was a helpful and necessary aid for the interpretation of the sensor data.

All participants took part in the telephone interviews (each one after T1 and T2); 4 persons were supported by relatives in both interviews.

There were no further problems in scheduling personal or telephone appointments or in the transfer of study documents and actigraphy to the study centre by the patients.

The telephone hotline was frequently used by the patients and their relatives before and during enrolment regarding organizational aspects (e.g., study duration and scheduling postponements) and mostly actigraphy (e.g., weight and size), indicating that this approach was feasible.

For further information and an overview of barriers and facilitators subdivided in all domains see Additional file 3.

##### Data collection in the clusters

All GPs submitted their completed questionnaires (the QCPC and evaluation forms for the training and the recruitment process), and for 91% of the patients (*n* = 20) the completed checklist as required.

The GPs frequently used the study centre hotline, mostly regarding recruitment but also to request additional recruitment documents.

Despite the commitment of all GPs, only five GPs attended on the agreed date, so one cluster was not represented. In the additional individual telephone interview about the recruitment procedure, one GP out of each practice took part.

Additional resources involved in the GPs’ study participation included personnel (office staff) and time; nevertheless, the GPs were well organized, so their study participation seemed to be integrated into their daily practice in an acceptable and practicable way.

For further information and an overview of barriers and facilitators subdivided in all domains see Additional file 3.

##### Data collection in the PTs

There were no problems with the PTs completing and submitting the standardized questionnaires. All PTs submitted the completed guides, and 85% the additional treatment documentation as required.

Individual telephone interviews with the PTs took place as planned.

Time expenditure and organizational efforts were limited, and study participation was reported to be easy to integrate into daily practice. The study centre hotline was mainly contacted regarding organizational issues (prescription filling, study procedures, and requests for informational and educational flyers).

Data collection (the DHI and miniBEST) was reported as feasible, as it was the delivery of these questionnaires by the patients and additional emails from the research team.

For further information and an overview of barriers and facilitators subdivided in all domains see Additional file 3.

### Feasibility of the intervention components and implementation strategy

#### The context: characteristics of the GP and PT practices

The GP practices treated over 500 to 2000 patients per quarter with 39% (mean) of patients being older than 60 years and an average of 33% (mean) of the patients having at least two chronic diseases.

The PT practices treated between fewer than 500 patients and more than 2000 patients per quarter (mode < 500 patients). On average, 57% of the patients were over 60 years old, and 47% had at least two chronic diseases.

During the intervention implementation, the following were reported as barriers for patients: low treatment adherence; a lack of awareness of the intervention impact; and visual, writing or comprehension problems. Social support by relatives was reported as a facilitator.

Regarding the health professionals’ motivation, positive expectations and familiarity with the intervention and support via the helpline were reported as facilitators. A lack of interdisciplinary exchange was rated as a barrier.

Organizational aspects (lack of time, short treatment units in the PT practices, and long waiting times for appointments with medical specialists/PTs) were rated as barriers. Intra-professional exchange was reported as a facilitator.

For further information and an overview of barriers and facilitators subdivided in all domains see Additional file 3.

#### Delivery to and response of clusters

All GPs took part in one of the offered training sessions in May/June and rated all statements regarding the achievement of the learning objectives as entirely true to partly true, indicating the good acceptance of the training. The GPs especially emphasized their satisfaction with the practical exercises, the good atmosphere and the small group size but requested the additional application of the checklist in a case study. All GPs believed that they had the competence to apply the checklist in practice. For further information about the results of the evaluation forms see Table 7.

**Table 7:** Evaluation of educational training of GPs

No.	Evaluation area and domain	1st educational training date	2nd educational training date	Total
		(*n* = 5)	(*n* = 2)	(*n* = 7)
Dissemination of knowledge, *median (range)*				
	At the training, I was systematically taught			
1	The differences between the most important vertigo syndromes.	2.0 (1.0–3.0)	1.5 (1.0–2.0)	2.0 (1.0–3.0)
2	Methods for diagnosing positional vertigo.	1.0 (1.0–1.0)	1.5. (1.0–2.0)	1.0 (1.0–2.0)
3	Forms of therapy and their instructions for the most important vertigo syndromes.	2.0 (1.0–4.0)	1.5. (1.0–2.0)	2.0 (1.0–4.0)
4	How to apply the checklist in practice.	1.0 (1.0–2.0)	1.0 (1.0–1.0)	1.0 (1.0–2.0)
Gain in know-how skills, *median (range)*				
5	At the training, I was systematically taught a neurological screening.	2.0 (1.0–3.0)	1.5 (1.0–2.0)	2.0 (1.0–3.0)
6	After the training, I feel able to apply the demonstrated examination techniques.	2.0 (1.0–2.0)	1.0 (1.0–1.0)	1.0 (1.0–2.0)
7	The contents of the training were adequate for the independent practical application of the checklist.	2.0 (1.0–2.0)	1.0 (1.0–1.0)	1.0 (1.0–2.0)
8	The workshop was well-structured and organized for practical application of the checklist.	2.0 (1.0–2,0)	1.0 (1.0–1.0)	1.0 (1.0–2.0)
Temporal organization, *median (range)*				
9	The duration of the workshop was appropriate.	1.5 (1.0–2.0)	1.0 (1.0–1.0)	1.0 (1.0–2.0)
Total quality of educational training (No 1–9), *mean (range)*		1.7 (1.0–2.0)	1.2 (1.0–1.5)	1.3 (1.0–2.0)
Other, *median (range)*				
10	In your opinion, is there a need for such training among GPs?	1.0 (1.0–2.0)	1.0 (1.0–1.0)	1.0 (1.0–2.0)
11	Do you already use the presented techniques for vertigo syndromes?	3.0 (1.0–4.0)	2.5 (2.0–3.0)	3.0 (1.0–4.0)

Coding: 1 = “entirely true”, 2 = “partly true”, 3 = “rather not true”, 4 = “completely untrue”

Missing values: Item 9 (*n* = 1)

*Note*: Besides these 11 domains, the following 3 questions could be answered in free text form (qualitative analysis): What did you particularly like about the training? What did you not like about the training? What else would you have liked?

Furthermore, the GPs asked for a brief summary of the whole examination procedure for patients with VDB in the form of a written handout with pictures or a homepage with videos.

The checklist was applied to 91% of the study participants (*n* = 20) at least once. The expectations of the participating GPs were not in line with the initial aim of the checklist. The GPs expected a more comprehensive guideline to patient history and diagnoses rather than a short checklist.

*“If the patient goes and says ‘He asked me three questions and then sent me to an otolaryngologist’, then he feels as usual that someone has not really taken him seriously and has not even examined him in a structured way.”* (GP, 45 years).

The GPs stated that a chronological structure with a more detailed patient history section would be preferable, e.g., a two-sided document to combine the patient history; examination; and outcomes, such as referrals. They rated the paper format of the checklist (210 mm × 297 mm, ISO DIN A4) as feasible, and one GP stated that a digital form would be too complicated and could not be used in daily practice.

According to the GPs, problems completing the checklist arose due to unclear instructions. Overall, the GPs completed the checklist rather incompletely and made partly incomplete entries; e.g., they did not note referral to physical therapy.

Further deviations from the intervention protocol occurred in the timing of checklist application. The GPs frequently first completed the checklist during recruitment, which results in the baseline assessment not being able to be performed prior to the intervention as intended. A total of 41% of the patients attended all GP appointments as required (initial diagnostics, and follow-up after 4 weeks, follow-up after 8 weeks/3 months), 14% were seen by their GP twice and 36% kept only the initial appointment. According to the GPs, the reasons for the patients not attending all appointments were the GPs forgetting to actively schedule patients for their next appointment at the practice, but mostly the patients’ poor adherence to the prescribed treatment schedule. The patients reported lack of scheduling by the GP, as most of them proactively contacted their GP due to the need for a follow-up referral to a PT. In two patients (9%) the checklist was not used at all.

A total of 14 patients (64%) were referred to physical therapy. For 79% of the patients, the GPs used a VDB-specific ICD code (3 missing) and for 71% the VDB-specific indication code (1 missing) was used as intended. Most GPs referred patients to physical therapy (*n* = 11, 79%; 3 missing), and for two patients (14%), the GP additionally prescribed classical therapeutic massage. Mostly, there was no interdisciplinary exchange between the GPs and PTs.

A total of 46% of the study participants received a referral to at least one medical specialist.

All GPs stated that the high time expenditure required to apply the checklist (range: 20–30 min) made an appointment outside office hours necessary. Routine was mentioned to be beneficial for the application of the checklist in daily practice.

*“If you do it [the checklist] more often, you can easily get it done in 15 to 20 minutes. […] And these are worthwhile 20 minutes [...]. So, you save a lot of time afterwards.”* (GP, 45 years).

Despite the required adaptations to the procedure to enhance its user-friendliness, the GPs saw added value because the standardized procedure gave them security in dealing with affected persons, and the exclusion of patients with alarm symptoms. This finding indicates a change in the GPs competence and behaviour in the treatment of patients with VDB.

Although all GPs appreciated the offered telephone helpline, only one GP used it for a question in completing the checklist (call duration < 5 min).

The GPs were pleased with the qualification certificate and the certificate for study participation, which some of them displayed in their practice.

For further information and an overview of barriers and facilitators subdivided in all domains see Additional file 3.

#### Delivery to and response of PTs

All PTs attended the educational training at the beginning of the study directly after recruitment of all participating PTs in May. All statements regarding the achievement of learning objectives were rated as entirely true, indicating a very good acceptance of the workshop. They especially highlighted the interplay between the theoretical and practical parts, and all PTs believed they had the competence to apply the guide in practice. For further information about the results of the evaluation forms see Additional file 4.

The PTs rated the supportive materials as helpful for understanding the content, whereas they requested further summaries of treatment techniques in written form or video tutorials.

The guide was applied to all study participants who were referred to trained PTs. The PTs evaluated the content and structure of the guide as good and rated the paper format (297 mm × 420 mm, ISO DIN A3) as feasible and clearly arranged. The time required for the application of the guide differed between the PTs (range: 15–30 min), and most managed to complete it within one treatment unit. There were no additional personal resources needed. Overall, the PTs completed the physical assessment section of the guide fully but used the performed assessments rather incompletely.

All PTs stated to have profited from the use of the guide, especially due to the structured procedure, which allowed the patients to benefit from adequate treatment and efficient clinical reasoning.

*“If I save time with the diagnostic process, he [the patient] has more time for therapy at the 1st appointment. […] If I know in a more focused way where exactly the problem is, I can help even better, offer support. […] So, I think he simply benefits from the fact that you know much more focused* (PT, 33 years)

Overall, the PTs rated the intervention as acceptable and feasible in daily practice, with practical exercise through repeated application of the guide leading to safety in use and thus to time savings.

The PTs reported changes in their competence and behaviour and indicated that their self-efficacy was strengthened by the knowledge and skills they acquired during the training.

The PTs adhered to the guide well so that all patients received VDB-specific treatment and at least one target group-oriented flyer (92%). The PTs evaluated the treatment as targeted to patient needs and age.

*“I always put a cross on the exercises that we have discussed or that they can or should do at home. And that simply makes it easier. There is the picture and the text, well explained. I find it very helpful.”* (PT, 52 years)

Most PTs reported that interdisciplinary interaction with the GPs was scarce, whereas intra-professional exchange in practice teams and with colleagues outside increased.

The utilization of the telephone helpline was scarce (1 call, call duration < 5 min). A reason for a lack of use of the helpline was stated only by one PT (forgot about the option).

For further information and an overview of barriers and facilitators subdivided in all domains see Additional file 3.

#### Delivery to and response of individuals

Almost all patients (91%) received the GP intervention between June 2019 and January 2020, and they were mostly satisfied with their treatment. A total of 10 patients (46%) received a referral to at least one medical specialist (cardiologist, ophthalmologist, neurologist or ENT physician) and 64% (*n* = 14) received a referral to physical therapy. However, 14% (*n* = 3) received neither a referral to PT nor a referral to a medical specialist. Additionally, two patients declined a referral to a PT due to lack of interest and focus on other acute health issues. The GPs reported patients’ characteristics (poor motivation and lack of awareness about the effects of specific therapy) as potential barriers for further referral, as well as organizational issues. 93% of the patients with referrals to a PT decided to go to practices with specially trained PTs and reported being satisfied with therapy. The patients rated the leaflets for home exercises as easy to understand and the exercises to be feasible to complete at home, whereas two persons received help from relatives in performing the exercises. Most reported that they performed the exercises regularly, motivated by the hope of symptom relief, but a few reported that they only sporadically performed exercises due to lack of time, a focus on other health issues or forgetting.

*“I just realized it is getting better. […] Vertigo seems to be a vicious circle. That means when I have vertigo, I do less activity. Less activity means, especially in older people, that the muscles weaken and the problem becomes increasingly worse. [...] So if I now try to at least do exercises and train these areas a little bit […] I hope that the strength, i.e., the intensity of the vertigo, is no longer the same as before.”* (Patient, 67 years)

#### Unintended consequences

Health professionals reported no unintended harmful consequences for patients or themselves of the application their parts of the intervention. No patients suffered harm, e.g., due to a fall event directly related to the intervention, which indicates its safety.

## Discussion

This study mainly confirmed the feasibility of the proposed intervention and study design but also identified aspects to be optimized.

We made use of reported promising recruitment strategies, such as personal contact [47, 48]; aimed to minimize the time demand for participants [47]; and provided payment [49]. Nevertheless, the recruitment of GPs was difficult, as reported in other studies [47, 49]. However, in contrast to these findings, we did not experience any dropouts during the study. In line with previous recommendations [48], we planned to involve practice staff in informing patients about the study. However, we observed that brief training and written guidelines would have been useful. In addition, we found that close contact between the research team and the GPs to identify problems early and misunderstandings might have led to the more efficient recruitment of patients. Additionally, even though the reported prevalence of VDB has been reported to be up to 50% in patients over 65 years [5–8], the identification of appropriate patients is difficult and cannot be explained by the characteristics of GP practices alone. We hypothesize that the frequently reported problem of diagnosing VBD, which favours extensive health care utilization [14, 15] might have led to that issue.

The recruitment of PTs was easier, but early contact seems to be advisable. In addition, more than a single PT per practice should be trained to both avoid long waiting times and optimize the reach of the intervention.

As the patients mostly opted for measurements in their homes, the need for study assistants should be calculated carefully. The engagement of relatives was found to facilitate patient adherence and attrition. We therefore suggest a stronger involvement of relatives, which is consistent with previous research [50].

Completing the IPAQ, which was developed to be used in a younger population [31], was challenging and resulted in many missing values, so its use in a larger trial is not recommended. The response rate and acceptance for both physical activity sensor models were high, but one (Move4) model provided better data; therefore, we recommend its use with an adapted version of the physical activity diary including standardized, quantitative dizziness assessment (e.g., DHI) for the evaluation of physical activity in future trials. For adequate interpretation of objective activity measures, patients should be classified according to their gait mobility (e.g. use of a walking aid) [51, 52]. In addition, we recommend a standardized gait test (100 m or 20 m) [51, 52] at the beginning of each measurement period for the evaluation of relevant gait parameters.

We used a combination of different implementation strategies according to the Expert Recommendations for Implementing Change [53]. In line with previous trials [54–58], all health professionals emphasized the training to be essential and appreciated the interlocking of the theoretical and practical parts [57, 59]. Since GPs mentioned that they were not sufficiently trained in the practical application of the checklist during the educational training, we plan to include the application of the checklist in a case study, for which a longer time period of training should be set. Since the PTs were interested in information about the GP tasks, joint training of both GPs and PTs, including an overlapping introduction, may be reasonable and might additionally have a positive impact on interdisciplinary communication. The use of supportive resources is well established as part of effective interventions [57] and the materials were positively received and used. For the main trial, the request for further summaries, e.g., in the form of a website with videos and written material, should be taken into account.

Although the intervention was delivered to health professionals as intended, it was not sufficiently delivered to the patients by the GPs, especially due to adherence issues in application of the checklist. In addition to time issues, the main reason for the lack of adherence in the application of the checklist was probably the GPs’ different expectations of the intervention compared to the initial aim of the developers. This deviation could be due to the small number of participants (at the development and feasibility phase), which may have led to distorted and non-generalizable opinions from overly motivated participants. We are confident that the GPs’ adherence to the intervention protocol could be improved through a combined application of a revised version of the checklist; more pronounced practical exercises; and improved supportive material related to diagnostic and therapeutic techniques, such as positioning manoeuvres. The compliance of GPs with planned timelines could be improved by using telephone reminders, which is a well-established approach [60]. The use of the PT guide was implemented as planned and was found to be feasible. Both the PTs and GPs rated the paper material as practicable, while some PTs reported that they would appreciate a digital form, provided that the form would be technically compatible with existing systems. For the main study, the option of a digital application was envisaged, but this option needs to be further evaluated in view of the preferences of the participants. However, the integration of interventions into practice software could offer the possibility to promote the fitting of interventions into daily practice [58] and may additionally improve interdisciplinary exchange [61].

Despite the health professionals’ enthusiasm for the telephone helpline, its utilization was low, and contact on a regular basis might be beneficial [62].

Our results show that the success of intervention also depends on patient adherence, which was mostly good in this study, e.g., in the regular performance of home exercises. Only a few patients showed a lack of adherence, which is a well-known problem in implementation of interventions [56, 63, 64]. Reasons for the well-known problem of lack of adherence [56, 63, 64] must be analysed individually to find solutions to promote acceptance and intervention implementation. Since we found that individual characteristics impacted the success of the intervention application, patients’ abilities and behaviour must be taken into account.

In contrast to the findings of the previous part of this study (development phase), which identified the wish for better multi-disciplinary exchange as a key to successful treatment of VDB, our results showed very low communication between the GPs and PTs. Since good multi-disciplinary communication and cooperation have been stated as facilitators by health professionals [56, 61, 65] and patients [50], it seems to be beneficial to invest more efforts to improve this communication.

Overall, this study confirmed that our programme activities were mainly effective in changing health professionals’ behaviour, as hypothesized in our logic model. Despite the initial difficulties, all health professionals used the new knowledge and skills to apply their part of the intervention, with some adjustments. They perceived an improvement in competence and self-efficacy, which contributed to the improvement in the patient’s situation.

There were no harmful unintended consequences of the intervention.

## Strengths and limitations

A strength of the study is the rigorous and comprehensive process evaluation in the feasibility stage, which is highly recommended for newly developed interventions [32], and the mixed-method approach considering different perspectives to achieve a detailed comprehension of how the intervention works [32].

Our study also has limitations, especially regarding problems in recruitment. Since the participants were difficult to recruit, only a small number of GPs and – consequently – patients were included, leading to a potential bias in the results. Notably, mainly younger and more physically active patients were enrolled in the study, whereas the intervention was initially targeted at older patients with multi-morbidity and immobility.

## Conclusion

Although the study results provide good support for the feasibility of the intervention in older patients with VDB in primary care, they reveal important insights into challenges and the need for improvement of the intervention, its implementation strategy and study procedures. In particular, the recruitment of GPs and patients is challenging, and more detailed guidance from the research team for GPs is required. Due to difficulties with GPs’ adherence to the study and intervention protocol, the intensification of regular exchange between the GPs and the research team is highly recommended to eliminate misunderstandings. Furthermore, a revision of the checklist is necessary. In a next step, the further developed and optimized intervention might be investigated for its effectiveness in a large cRCT.

## Supplementary Information

**Additional file 1.** Manual for the recruitment of patients

**Additional file 2.** Overview of components and methods of the process evaluation alongside the feasibility study (based on Logic model, study process and domains by Grant et al. [33])

**Additional file 3.** Barriers and facilitators alongside the feasibility study

**Additional file 4.** Evaluation of educational training of PTs

## Data Availability

All data generated or analysed and the measurements used during this study not included in this report, are available from the authors on request.

## Abbreviations

BCW: Behaviour Change Wheel
CONSORT: Consolidated Standards of Reporting Trials
COM-B: Capability-Opportunity-Motivation-Behaviour (model)
CPW: Care pathway
cRCT: Cluster randomized controlled trial
CRD: Centre for Reviews and Dissemination
DHI: Dizziness Handicap Inventory
ENT: Ear-nose-throat
EQ-5D-5L: EuroQol5-dimension 5-level
GP: General practitioner
ICD: International Statistical Classification of Diseases and Related Health Problems
IPAQ: International Physical Activity Questionnaire
MET-min/week: Metabolic equivalent task minutes per week
miniBEST: Mini-Balance Evaluation Systems Test
MRC: Medical Research Council
PT: Physical therapist
QCPC: Questionnaire of Chronic Illness Care in Primary Care
REDCap: Research Electronic Data Capture
TIDieR: Template for Intervention Description and Replication
UK: United Kingdom
VAS: Visual analog scale
VDB: Vertigo, dizziness and balance disorders
