# Supplementary material for: Improving mobility and participation of older people with vertigo, dizziness and balance disorders in primary care using a care pathway: feasibility study and process evaluation
Source: BMC Fam Pract. 2021 Apr 2;22:62. doi: 10.1186/s12875-021-01410-2 (PMC8017844; doi:10.1186/s12875-021-01410-2)
Supplement: Supplementary file 1 — Additional file 1. Manual for the recruitment of patients [file 12875_2021_1410_MOESM1_ESM.docx]

## Additional file 1 Manual for the recruitment of patients

**Manual for the recruitment of patients**

Please note the following inclusion and exclusion criteria for the recruitment of eligible study participants from your patient master:

| **Inclusion criteria** | **Exclusion criteria** |
| --- | --- |
| - patients **≥65 years** - consultation to you **within the last three years** due to vertigo, dizziness or balance disorders - **walking ability** of 10 metres (with or without walking aids) | - **insufficient verbal and cognitive command** of the German language to give written informed consent, complete questionnaires and follow verbal and written instructions - serious conditions or diseases requiring in-patient **hospital treatment** - affected patients with **legal guardian** |

**ICD-10-Codes:** Selection of relevant diagnoses according to their described frequency*

R42 (dizziness and giddiness)

G62 (other and unspecified polyneuropathies)

H81 (disorders of vestibular function)

F45.8 (other somatoform disorders)

I95.1 (orthostatic hypotension)

G63 (polyneuropathy in diseases classified elsewhere)

G43.1 (migraine with aura)

R26 (without R26.1) (abnormalities of gait and mobility)

* Rieger A., Mansmann U., Maier W., Seitz L., Brandt T, Strupp M. et al. Management of patients with the cardinal symptom dizziness or vertigo. Gesundheitswesen. 2014 76(6):e32-8. doi: 10.1055/s-0033-1357145.
