# Supplementary material for: Improving mobility and participation of older people with vertigo, dizziness and balance disorders in primary care using a care pathway: feasibility study and process evaluation
Source: BMC Fam Pract. 2021 Apr 2;22:62. doi: 10.1186/s12875-021-01410-2 (PMC8017844; doi:10.1186/s12875-021-01410-2)
Supplement: Supplementary file 2 — Additional file 2. Overview of components and methods of the process evaluation alongside the feasibility study (based on Logic model, study process and domains by Grant et al. [33]) [file 12875_2021_1410_MOESM2_ESM.docx]

## Additional file 2 Overview of components and methods of the process evaluation alongside the feasibility study (based on Logic model, study process and domains by Grant et al. [33])

| **Aim** | **Domain** | **Research questions** | **Evaluation focus area** | **Data collection methods and measures** | **Respondents** | | | | **Stage of study** |
| --- | --- | --- | --- | --- | --- | --- | --- | --- | --- |
|  |  |  |  |  | Research team | GPs | PTs | Patients |  |
| Trial feasibility | Recruitment of clusters* and PTs | How were health professional practices sampled and recruited? | Evaluation of the recruitment procedure, including required resources and difficulties | Field notes by the research team | X |  |  |  | Prior to and during the intervention |
|  |  | How did health professional practices respond to the recruitment approach? | Satisfaction with the recruitment approach and study documents | Group interview with GPs |  | X |  |  |  |
|  |  |  |  | Individual interviews with PTs |  |  | X |  |  |
|  |  |  |  | Field notes by the research team | X |  |  |  |  |
|  |  | Who agreed to participate? | Characteristics of the participants | Standardized questionnaire on sociodemographic and structural practice data based on the QCPC |  | X | X |  |  |
|  |  | Why did they agree to participate? | Reasons for participation | Group interview with GPs |  | X |  |  |  |
|  |  |  |  | Individual interviews with PTs |  |  | X |  |  |
|  |  | Why did they not agree to participate? | Reasons for non-participation | Field notes by the research team | X |  |  |  |  |
|  |  | What was the ability to retain participants throughout the follow-up period? | Number of missing data: number of withdrawals and drop-outs including reasons | Field notes by the research team | X |  |  |  |  |
|  | Recruitment and reach of individuals* | How were individuals sampled and recruited? | Evaluation of the recruitment procedure, including required resources and difficulties | Group interview with GPs |  | X |  |  |  |
|  |  |  |  | Individual interview with GPs |  | X |  |  |  |
|  |  |  |  | Standardized questionnaire on the recruitment process |  | X |  |  |  |
|  |  |  |  | Field notes by the research team | X |  |  |  |  |
|  |  |  |  | Field notes on contact with GPs via telephone or email |  | X |  |  |  |
|  |  | How did the individuals respond to the recruitment approach? | Satisfaction with the recruitment approach and study documents | Individual interviews with patients |  |  |  | X |  |
|  |  |  |  | Group interview with GPs |  | X |  |  |  |
|  |  |  |  | Individual interview with GPs |  | X |  |  |  |
|  |  | Who agreed to participate?  Are they representative? | Characteristics of participants | Standardized questionnaire on sociodemographic data |  |  |  | X |  |
|  |  | Why did individuals agree to participate? | Reasons for participation | Individual interviews with patients |  |  |  | X |  |
|  |  |  |  | Group interview with GPs |  | X |  |  |  |
|  |  | Why did individuals not agree to participate? | Reasons for non-participation | Patients’ cancellation forms |  |  |  | X |  |
|  |  |  |  | Group interview with GPs |  | X |  |  |  |
|  |  | What was the ability to retain participants throughout the follow-up period? | Number of missing data: number of withdrawals and drop-outs including reasons | Field notes by the research team | X |  |  |  |  |
|  | Outcome measures and data collection procedures | Were the data collection procedures and outcome measures delivered as intended?  How was the data collection procedures adopted?  Were there unintended changes in data collection procedures? | Evaluation of the performance of data collection procedures and outcome measures  Study assistants’ adherence to the measurement protocol, including difficulties and adaptions  Feasibility of outcome measures  Feasibility of organizational issues like scheduling | Field notes by the study assistant after each measurement appointment | X |  |  |  | During the intervention |
|  |  |  |  | Field notes by the research team | X |  |  |  |  |
|  |  |  | Feasibility and preference of the setting options | Field notes by the study assistant after each measurement appointment | X |  |  |  |  |
|  |  |  | Amount of missing data | Field notes by the research team | X |  |  |  |  |
|  |  | How did the target population respond?  Were there differences between the individuals? | Acceptability of questionnaires, performance tests, physical activity diary, and actigraphy devices, including preferences for one approach | Standardized evaluation forms after each questionnaire |  |  |  | X |  |
|  |  |  |  | Field notes on contact with patients via telephone or email |  |  |  | X |  |
|  |  |  |  | Field notes by the research team | X |  |  |  |  |
|  |  |  |  | Individual interviews with patients |  |  |  | X |  |
|  |  |  | Satisfaction with the study organization and the effort of study participation  Satisfaction with personal/virtual appointments  Willingness to conduct interviews  Satisfaction with the support by the study team | Field notes by the patients^1^ |  |  |  | X |  |
|  |  |  |  | Field notes by the research team | X |  |  |  |  |
|  |  |  |  | Individual interviews with patients |  |  |  | X |  |
|  |  |  |  | Field notes on contact with patients via telephone or email |  |  |  | X |  |
|  |  |  | Acceptability and preference of the setting options | Field notes by the study assistant after each measurement appointment | X |  |  |  |  |
|  |  |  |  | Individual interviews with patients |  |  |  | X |  |
|  |  | How did the GPs and PTs respond?  Were there differences between the clusters? | Satisfaction with the study organization and the effort of study participation  Feasibility of personal/virtual appointments  Willingness to conduct interviews  Integrability of participation in daily practice | Field notes on contact with health professionals via telephone or email |  | X | X |  |  |
|  |  |  |  | Field notes by the research team | X |  |  |  |  |
|  |  |  |  | Group interview with GPs |  | X |  |  |  |
|  |  |  |  | Individual interviews with PTs |  |  | X |  |  |
| Feasibility of the intervention components and implementation strategy | Context* | In what context was the intervention implemented? | Structural characteristics of GP and PT practices | Standardized questionnaire on sociodemographic and structural practice data based on the QCPC |  | X | X |  | Prior to the intervention |
|  |  | How did contextual factors influence the implementation process? | Contextual factors as barriers/facilitators in implementing the intervention | Group interview with GPs |  | X |  |  | During the intervention |
|  |  |  |  | Individual interviews with PTs |  |  | X |  |  |
|  |  |  |  | Individual interviews with patients |  |  |  | X |  |
|  |  |  |  | Field notes by the research team | X |  |  |  |  |
|  | Delivery to clusters* and PTs | What intervention was delivered to each cluster/PT practice?  Were the components of the intervention introduced as planned? | Evaluation of the educational trainings, including supportive materials  Evaluation of the telephone helplines  Evaluation of the importance of qualification certificate and certificate for study participation | Field notes by the research team | X |  |  |  | During the intervention |
|  |  |  |  | Group interview with GPs |  | X |  |  |  |
|  |  |  |  | Individual interviews with PTs |  |  | X |  |  |
|  |  |  |  | Field notes on contact with health professionals via telephone or email |  | X | X |  |  |
|  | Response of clusters* and PTs | How were the GPs’ and PTs’ perceptions of the intervention and uptake? | Satisfaction with the educational trainings, including supportive materials | Standardized evaluation forms for the educational trainings |  | X | X |  | During and after the intervention |
|  |  |  |  | Group interview with GPs |  | X |  |  |  |
|  |  |  |  | Individual interviews with PTs |  |  | X |  |  |
|  |  |  |  | Field notes on contact with health professionals via telephone or email |  | X | X |  |  |
|  |  |  |  | Field notes by the research team | X |  |  |  |  |
|  |  |  | Attendance in educational trainings | Field notes by the research team | X |  |  |  |  |
|  |  |  | Satisfaction with the telephone helplines  Use of the telephone helplines  Fulfilment of expectations regarding the intervention | Group interview with GPs |  | X |  |  |  |
|  |  |  |  | Individual interviews with PTs |  |  | X |  |  |
|  |  |  |  | Field notes on contact with health professionals via telephone or email |  | X | X |  |  |
|  |  |  |  | Field notes by the research team | X |  |  |  |  |
|  |  |  | Satisfaction with the checklist/guide as intervention  Number and type of supportive material distributed by the PTs to the patients | Group interview with GPs |  | X |  |  |  |
|  |  |  |  | Individual interviews with PTs |  |  | X |  |  |
|  |  |  |  | Field notes on contact with health professionals via telephone or email |  | X | X |  |  |
|  |  |  |  | Field notes by GPs^2^ |  | X |  |  |  |
|  |  |  |  | Field notes by PTs^3^ |  |  | X |  |  |
|  |  | How was the intervention adopted by the GPs and PTs? | GPs’ and PTs’ adherence to the recommendations of the checklist/guide | Group interview with GPs |  | X |  |  |  |
|  |  |  |  | Individual interviews with PTs |  |  | X |  |  |
|  |  |  |  | Field notes on contact with health professionals via telephone or email |  | X | X |  |  |
|  |  |  |  | Field notes by the research team | X |  |  |  |  |
|  |  |  |  | Field notes by GPs^2^ |  | X |  |  |  |
|  |  |  |  | Field notes by PTs^3^ |  |  | X |  |  |
|  |  |  | Deviations from implementation protocol | Field notes by the research team | X |  |  |  |  |
|  |  | How were the GPs’ and PTs’ experiences and perceived changes in attitude and behaviour?  Were there any changes in daily practice? | Attitude regarding intervention *(e.g., usefulness in daily practice)*  Behavioural change/feeling of security/self-efficacy  Integration of intervention into daily practice | Group interview with GPs |  | X |  |  |  |
|  |  |  |  | Individual interviews with PTs |  |  | X |  |  |
|  |  |  |  | Field notes on contact with health professionals via telephone or email |  | X | X |  |  |
|  |  |  |  | Field notes by the research team | X |  |  |  |  |
|  |  | What were the facilitators and barriers for a successful implementation? | Experiences regarding barriers/facilitators of GPs and PTs during the implementation process | Group interview with GPs |  | X |  |  |  |
|  |  |  |  | Individual interviews with PTs |  |  | X |  |  |
|  |  |  |  | Field notes by the research team | X |  |  |  |  |
|  | Delivery to individuals* | What intervention was delivered to individuals?  Who received the intervention?  Were the components of the intervention introduced as planned? | Evaluation of the treatment by GPs according to the checklist  Evaluation of the treatment by PTs according to the guide, including the distribution of supportive materials | Individual interviews with patients |  |  |  | X | During and after the intervention |
|  |  |  |  | Group interview with GPs |  | X |  |  |  |
|  |  |  |  | Individual interviews with PTs |  |  | X |  |  |
|  |  |  |  | Field notes by the research team | X |  |  |  |  |
|  |  |  |  | Field notes on contact with patients via telephone or email |  |  |  | X |  |
|  |  |  |  | Field noted by GPs^2^ |  | X |  |  |  |
|  |  |  |  | Field notes by PTs^3^ |  |  | X |  |  |
|  | Response of individuals* | How did the target population respond to the instructions of the GPs and PTs?  Were there any differences between the individuals? | Satisfaction with the treatment by GPs  Satisfaction with the treatment by PTs, including supportive materials | Individual interviews with patients |  |  |  | X | During the intervention |
|  |  |  |  | Field notes by the patients^1^ |  |  |  | X |  |
|  |  |  |  | Field notes on contact with patients via telephone or email |  |  |  | X |  |
|  |  | Were there any adoptions?  Were there differences between the individuals? | Adherence to recommendations of GPs  Adherence to recommendations of PTs, including home exercises | Individual interviews with patients |  |  |  | X |  |
|  |  |  |  | Group interview with GPs |  | X |  |  |  |
|  |  |  |  | Individual interviews with PTs |  |  | X |  |  |
|  |  | How were individuals’ experiences and perceived changes in attitude and behaviour?  Were there any changes in daily life? | Behavioural change/feeling of security/self-efficacy  Integration of programme activities into daily practice | Individual interviews with patients |  |  |  | X |  |
|  | Unintended consequences* | Were there unintended changes in processes and outcomes? | Unintended consequences (harmful/beneficial) for patients | Individual interviews with patients |  |  |  | X | During the intervention |
|  |  |  |  | Field notes on contact with patients via telephone or email |  |  |  | X |  |
|  |  |  |  | Field notes by the research team | X |  |  |  |  |
|  |  |  | Unintended consequences (harmful/beneficial) for GPs and PTs | Group interview with GPs |  | X |  |  |  |
|  |  |  |  | Individual interviews with PTs |  |  | X |  |  |
|  |  |  |  | Field notes on contact with patients via telephone or email |  |  |  | X |  |
|  |  |  |  | Field notes by the research team | X |  |  |  |  |
| GP=general practitioner, PT=physical therapist, QCPC=Questionnaire of Chronic Illness Care in Primary Care  cluster=GP practice (here: one general practitioner as representative of the practice each); individuals=patients  * Domains by Grant et al. [33]  ^1^ Patients’ field notes in free text option in physical activity diary  ^2^ GPs’ field notes in form of a completed checklist including a free text option  ^3^ PTs’ field notes in form of a completed guide including a free text option and treatment documentation | | | | | | | | | |
