# Supplementary material for: Improving mobility and participation of older people with vertigo, dizziness and balance disorders in primary care using a care pathway: feasibility study and process evaluation
Source: BMC Fam Pract. 2021 Apr 2;22:62. doi: 10.1186/s12875-021-01410-2 (PMC8017844; doi:10.1186/s12875-021-01410-2)
Supplement: Supplementary file 3 — Additional file 3. Barriers and facilitators alongside the feasibility study [file 12875_2021_1410_MOESM3_ESM.docx]

## Additional file 3 Barriers and facilitators alongside the feasibility study

| **Aim** | **Domains*** | **Subdomain** | **Barriers** | **Facilitators** *(including recommendations)* |
| --- | --- | --- | --- | --- |
| Trial feasibility | Recruitment of clusters* | Recruitment procedure | - Limited availability and accessibility of GPs (research team) | - Use of fax instead of an email to submit information to GPs (GPs, research team) - Personal information about the project in practice on site instead of an information event at study centre (GPs, research team) - Distribution of complete and good structured information documents informing about the study content (GPs) |
|  |  | Willingness to participate | - Lack of time and interest in the study subject (GPs) | - GPs’ interest in the study subject and in research projects, positive expectations regarding the intervention (improvement of treatment quality through a structured approach and intra-professional exchange) (GPs) |
|  | Recruitment of PTs | Recruitment procedure | - Short period of time between the request and the start of the study (PTs) | - Invitation to take part in the study via telephone call followed by an email with further information (PTs, research team) |
|  |  | Willingness to participate | - Lack of time and interest in the study subject (PTs) | - PTs interest in the study subject, in educational trainings and in research projects, positive expectations regarding the intervention (improvement of treatment quality and security through a structured approach) (PTs) |
|  | Recruitment and reach of individuals* | Recruitment procedure | - The GPs’ uncertainty regarding the timing and protocol of the intended recruitment process (GPs) - Difficulties in identifying the appropriate target group (GPs) | - Individual telephone call with a research team member to repeat and clarify the intended recruitment process (GPs _recommendation_) - Additional time for clarification regarding the study organization in the educational training session (GPs _recommendation_) |
|  |  |  | - Difficulties in using the practice software for the identification of potential participants due to lack of time and equipment, and deficient entries in the practice software (GPs) | - Recruitment in personal contact between the GPs and patients (GPs, patients) - Support in recruitment by practice staff (GPs) |
|  |  |  | - Forgetting about the study and the recruitment in daily practice (GPs) | - Brief written summary of the study as a conversation aid and reminder for GPs (GPs _recommendation_) - Study folder for GPs to collect all study documents to support the organization of study participation (GPs _recommendation_) |
|  |  |  | - Lack of correspondence of patients in practice to inclusion criteria, e.g., due to cognitive impairment (GPs) - Unfavourable recruitment period, e.g., due to holiday season (GPs) |  |
|  |  | Information documents for patients | - Restricted readability for patients with visual impairment (patients) - Comprehension problems regarding the content of study documents (GPs) - Bureaucratic confusion and overload due to the quantity and extent of the study documents (research team) | - Clarification of study content and documents in personal contact between patients and their GP (GPs _recommendation_) - Additional document summarising the most important study information (GPs _recommendation_) |
|  |  | Knowledge about participation | - Lack of or delayed feedback from patients/study centre on confirmation/refusal of patients to participate (GPs) | - Continuous exchange between the GPs and the study centre regarding participating patients (GPs _recommendation_) |
|  |  | Willingness to participate | - Lack of time, poor health status and low level of suffering under VDB (patients) - High expenditure of time, work overload in study participation, concerns about devices and patients’ acceptance of their VDB symptoms as given and unchangeable (GPs) | - High level of personal psychological strain due to VDB (GP, patients) - Patients positive expectations regarding the intervention (improvement of their own situations or those of others, and general interest in study participation) and good experience regarding participation in studies (patients) |
|  | Outcome measures and data collection procedures | Organization of measurement appointments | - Non-attendance of the patients at the agreed time (research team) | - Written or telephone reminder for measurement appointments (research team _recommendation_) |
|  |  |  | - Physical therapy and measurement appointment at the same day (patients) | - Measurement in the patients homes (patients)/in study centre due to time savings (research team) - Social contact in personal meetings (patients) |
|  |  |  | - Problems in the realization of the intended intervals between the measurement points due to organizational issues (research team) |  |
|  |  |  | - Patients’ difficulties in distinguishing the responsibilities and tasks of the study team and their GPs (patients, research team) |  |
|  |  | Questionnaires | - Patients’ inability in completing the questionnaires independently due to visual impairment, writing difficulties or comprehension problems (patients, research team) | - Support in completing the questionnaires by the patients’ relatives or the study assistant (patients, research team) |
|  |  |  | - Forgetting to submit the completed questionnaires to study centre (research team) | - Telephone reminder to send completed questionnaires to study centre (research team) |
|  |  | Performance test | - Influence of the patients condition on a particular day and physical handicaps on the feasibility in performing the test (patients, research team) - Feeling of insecurity in performing the test (patients) - Spatial situation, e.g., potential stumbling blocks in the patients homes (research team) - Higher expenditure of time needed for the test performance in the patients homes (research team) | - Safety of performance due to the study assistants’ basic qualifications and competence (research team) - Explanations prior to and during the performance test (patients) - Sympathy to the study assistant conducting the performance test (research team) - Brief and understandable feedback on the results of the performance test (patients) |
|  |  | Actigraphy devices | - Concerns about and reluctance to devices (patients, research team) | - Detailed explanation and introduction in the use of the devices (research team _recommendation_) - Devices requiring low patient compliance (research team) - Practical handling of devices (research team) - Short intended period of wearing the devices, and possibility of depositing the devices temporary (patients, research team) |
|  |  |  | - Forgetting to submit the devices after measurement to study centre (research team) | - Telephone reminder to submit the devices to study centre (research team) |
|  |  |  | - Occurrence of technical problems favouring faulty and incomplete recordings (research team) |  |
|  |  |  | - Unintended consequences as sliding down, itching, skin irritations and mild oedema (patients) |  |
|  |  | Physical activity diary | - Patients’ inability in completing the activity diary independently due to visual impairment, writing difficulties or comprehension problems (patients, research team) | - Support in completing the activity diary by patients’ relatives (patients, research team) |
|  |  |  | - Forgetting to submit the completed activity diary to study centre (research team) | - Telephone reminder to submit the completed activity diary to study centre (research team) |
|  |  |  | - High expenditure of time to complete the activity diary favouring insufficient and estimated data (patients) |  |
|  |  | Interviews | - Strenuous for patients with hearing problems (patients) | - Support by the patients’ relatives in interviews (patients, research team) |
|  |  | Telephone hotline |  | - Continuous and low-threshold availability of research team members via telephone (GPs, patients, PTs, research team) |
| Feasibility the of intervention components and implementation strategy | Context* | Personal factors | - Patients’ low treatment adherence, lack of motivation and awareness of the interventions impact (GPs) - Visual impairment, writing or comprehension problems of patients (GPs, patients, research team) - Multifactorial aetiology of VDB (GPs, PTs) | - Positive expectations regarding the interventions effectiveness, motivation and high level of suffering (GPs, patients) - Social support by patients’ relatives (GPs, patients, PT, research team) |
|  |  |  | - Lack of interdisciplinary exchange between health professionals (GPs, PTs) | - Health professionals’ motives, motivation, positive expectations regarding the intervention (GPs, PTs) - Health professional experience/skills (familiarity with the intervention due to routine) (GPs, PTs) |
|  |  |  |  | - Conscious, low-threshold social support via telephone helplines (GPs, PTs, research team) |
|  |  | Organizational factors | - Lack of time in daily practice (GPs, PTs) - Short treatment units in PT practices (PTs) - Long waiting times for appointments with medical specialists/PTs (GPs, patients, PTs) - PT practices previously attributing less importance and time to diagnostics (PTs) | - Intra-professional exchange between the health professionals (PTs) |
|  | Delivery to and response of clusters* | Educational group training | - Lack of practical training in the application of the checklist (GPs) | - Practical training: Application of the checklist in a case study (GPs _recommendation_) - Combination of theoretical and practical training parts (GPs, research team) |
|  |  |  | - Timeslot between the educational training and the application of the checklist in practice (GPs) | - Repetition of the educational training (GPs _recommendation_) |
|  |  |  | - Undertimed slot for information about the intended study procedure (timelines, tasks, and aims of the intervention components) (GPs) | - Scheduled time for information about the intended study procedure (research team) - Separation of professional training and information about the intended study procedure (GPs) |
|  |  |  | - Short temporal duration of the training session (GPs, research team) | - Good atmosphere (GPs) - Small group size (GPs) - Qualification certificate (GPs) |
|  |  | Additional supportive materials |  | - Distribution of additional materials about training content (GPs) - Brief summary of examination procedures in form of a written handout with pictures or videos with examples on a homepage (GPs _recommendation_) - Certificate for study participation (GPs) |
|  |  | Content and structure of the checklist | - Deviations in the GPs’ expectations regarding the checklist compared to those of the developers (GPs, research team) - Uncertainty regarding the aim of checklist (GPs) | - Interventions good fit into demands in daily practice: Guideline character of the checklist for patient history and diagnoses in chronological and detailed order (GPs _recommendation_) - Paper material and practicable layout of the checklist (GPs) - Standardized procedure promoting the GPs’ and the patients’ feeling of security (GPs) |
|  |  | Completion and application of the checklist | - Unclear instructions for the completion of the checklist (GPs) | - Uniform formalities (clear definitions and instructions) (GPs _recommendation_) |
|  |  |  | - Uncertainty regarding the intended timing of the checklist application (GPs, research team) | - Individual telephone call with a research team member to repeat and clarify the intended timing of the checklist application (GPs, research team) |
|  |  |  | - Difficulties in arranging follow-up appointments as intended due to the patients’ poor adherence, forgetting in daily practice (GPs) or no active scheduling of the patients for their next appointment at the practice by the GP (patients) | - Telephone reminder for the patients by the GP practice to make an appointment (GPs _recommendation_) |
|  |  |  | - High expenditure of time for the application of the checklist (GPs) | - Routine in the use of the checklist in daily practice (GPs) |
|  |  | Referrals | - Long waiting times for appointments with medical specialists (GPs, patients) | - Personal contact of GPs with medical specialists in order to get an earlier appointment (GPs) |
|  |  |  |  | - Opportunity to give advice for special trained PTs (GPs) |
|  |  | Interdisciplinary exchange | - Lack of interdisciplinary exchange despite of the request of a therapy report (GPs) |  |
|  |  | Telephone helpline |  | - Continuous and low-threshold availability of experts via telephone (GPs, research team) |
|  | Delivery to and response of PTs | Educational group training | - Timeslot between the educational training and the practical application of guide (PTs) | - Repetition of the educational training (PTs _recommendation_) |
|  |  |  | - Short duration of training session (PTs, research team) | - Interlocking of theoretical and practical training parts (PTs, research team) - Practical training in completition of the guide (PTs, research team) - Qualification certificate (PTs) - Joint training date of both health professional groups, including an overlapping introduction of the study contents (PTs, research team _recommendation_) |
|  |  | Additional supportive materials |  | - Distribution of additional materials about training content (PTs) - Brief summary of treatment techniques in written form or video tutorials (PTs _recommendation_) - Certificate for study participation (PTs) |
|  |  | Content and structure of the guide |  | - Clarity through a coloured system (PTs) - Paper material and practicable layout of the guide (PTs) - Digital form of the guide with technical compatibility at the workplace (PTs _recommendation_) - Standardized procedure promoting the PTs’ feeling of security and the patients’ adequate treatment (PTs) |
|  |  | Completion and application of the guide | - Difficulties in classification of vertigo due to multifactorial aetiology (PTs) | - Free text option for the patient history (PTs _recommendation_) |
|  |  |  | - High expenditure of time for the application of the guide including pre- and postprocessing (PTs) | - Routine in the use of the guide in daily practice (PTs) - Time savings in patient history due to the standardized procedure and familiarity with the guide (PTs) - Reduced quantity of diagnostic tests (PTs _recommendation_) |
|  |  | Target group-oriented leaflets for home exercises |  | - Availability of target group-oriented flyer tailored to the patients’ needs and age (PTs) - Explanations and practical instructions of the exercises and highlighting of the corresponding picture instructions of the flyers (PTs) - Repetitions of the exercises and involvement of relatives favouring the patients’ compliance (PTs) |
|  |  | Interdisciplinary exchange | - Lack of interdisciplinary exchange despite the need for it (PTs) | - Good relationship between the GPs and PTs (PTs) |
|  |  | Intra-professional exchange |  | - Increased exchange within the practice team and with colleagues about knowledge and skills (PTs) |
|  |  | Other |  | - Group training for patients with VDB (PTs _recommendation_) |
|  |  | Telephone helpline |  | - Continuous and low-threshold availability of experts via telephone (PTs, research team) |
|  | Delivery to and response of individuals* | Diagnostic screening based on the checklist | - Patients’ feeling of insecurity in performing of tests (patients) | - Patients’ feeling that the health issue was sufficiently recorded (patients) |
|  |  | Referrals to medical specialist/PTs | - Patients’ low adherence to recommended referrals due to their lack of motivation and awareness of the positive impact of physical therapy on VDB (GPs) - Organizational issues (GPs) - Long waiting times for appointments with medical specialists/PTs (patients) |  |
|  |  | Physical therapy |  | - Physical therapy tailored to the patients’ age and needs (patients) |
|  |  | Target group-oriented leaflets |  | - Availability of flyer tailored to the target population (patients) - Easy to understand content and structure using a plain language (patients) |
|  |  | Home exercises | - Lack of time, focus on other more restrictive health conditions, and forgetting about the exercises in daily life favoured a sporadically performance of exercises (patients) | - Positive expectations regarding the effectiveness of exercises (hope of symptom relief) (patients) |
|  |  |  |  | - Practicability of exercises in the patients homes (patients) |
|  |  |  |  | - Support by relatives in the performance of exercises (patients) - Familiarity with the exercises (patients) - Use of aids like solid footwear (patients) |
|  | Unintended consequences* | Unintended harmful/beneficial consequences |  | - No unintended consequences (GPs, patients, PTs, research team) |
| GP=general practitioner, PT=physical therapist, VDB=vertigo, dizziness and balance disorders  cluster=GP practice; individuals=patients  * Domains by Grant et al. [33]  **Database:** statements from the research team based on: field notes; statements from participating GPs based on: a group interview, individual interviews, standardized questionnaires on recruitment process, standardized questionnaires on sociodemographic and structural practice data, standardized evaluation forms for the educational trainings, contact via telephone or email, field notes; statements from participating PTs based on: individual interviews, standardized questionnaire on sociodemographic and structural practice data, standardized evaluation forms for the educational training, contact via telephone or email, field notes; statements from participating patients based on: individual interviews, cancellation forms, standardized questionnaires on sociodemographic data, contact via telephone or email, standardized evaluation forms after each questionnaire, field notes | | | | |
