# Supplementary material for: Improving mobility and participation of older people with vertigo, dizziness and balance disorders in primary care using a care pathway: feasibility study and process evaluation
Source: BMC Fam Pract. 2021 Apr 2;22:62. doi: 10.1186/s12875-021-01410-2 (PMC8017844; doi:10.1186/s12875-021-01410-2)
Supplement: Supplementary file 4 — Additional file 4. Evaluation of educational training of PTs [file 12875_2021_1410_MOESM4_ESM.docx]

## Additional file 4 Evaluation of educational training of PTs

| No | **Evaluation area and domain** | | **Total** (n=11) |
| --- | --- | --- | --- |
| Dissemination of knowledge, *median (range)* | | |  |
|  | In the training, I was systematically taught | |  |
| 1 |  | the most important forms of vertigo. | 1.0 (1.0 – 1.0) |
| 2 |  | procedures for the assessment of findings. | 1.0 (1.0 – 1.0) |
| 3 |  | therapeutic approaches. | 1.0 (1.0 – 2.0) |
| 4 |  | how to apply the guide in practice. | 1.0 (1.0 – 1.0) |
| Gain in know-how/skills, *median (range)* | | |  |
| 5 | After the training, I feel able to apply the demonstrated examination techniques. | | 1.0 (1.0 – 3.0) |
| 6 | After the training, I feel able to apply the demonstrated treatment techniques. | | 1.0 (1.0 – 3.0) |
| 7 | The contents of the training were adequate for the independent practical application of the guide. | | 1.0 (1.0 – 2.0) |
| 8 | The training was well-structured and organized for practical application of the guide. | | 1.0 (1.0 – 2.0) |
| Temporal organization, *median (range)* | | |  |
| 9 | The duration of the workshop was appropriate. | | 1.0 (1.0 – 3.0) |
| Total quality of educational training (No 1 – 9), *mean (range)* | | | 1.0 (1.0 – 1.0) |
| Other, *median (range)* | | |  |
| 10 | In your opinion, is there a need for such training for PTs working in an outpatient setting? | | 1.5 (1.0 – 2.0) |
| 11 | Have you already applied the presented techniques for vertigo patients in your practice before the training? | | 3.0 (1.0 – 4.0) |
| Coding: 1="entirely true", 2="partly true", 3="rather not true", 4="completely untrue"  Missing values: Item 5 (n=1)  Not applicable: Item 10 (n=1)  *Note: Besides these 11 domains, the following 3 questions could be answered in free text form (qualitative analysis): What did you particularly like about the training? What did you not like about the training? What else would you have liked?* | | | |
